# Anaemia in Indians aged 10–19 years: Prevalence, burden and associated factors at national and regional levels

**DOI:** 10.1111/mcn.13391

**Published:** 2022-06-20

**Authors:** Samuel Scott, Anwesha Lahiri, Vani Sethi, Arjan de Wagt, Purnima Menon, Kapil Yadav, Mini Varghese, William Joe, Sheila C. Vir, Phuong Hong Nguyen

**Affiliations:** ^1^ International Food Policy Research Institute New Delhi India; ^2^ MRC Epidemiology Unit, Institute of Metabolic Science University of Cambridge School of Clinical Medicine Cambridge UK; ^3^ Population Research Centre, Institute of Economic Growth New Delhi India; ^4^ Nutrition Division, UNICEF New Delhi India; ^5^ Centre for Community Medicine, All India Institute of Medical Sciences New Delhi India; ^6^ Nutrition International New Delhi India; ^7^ Public Health Nutrition and Development Centre New Delhi India; ^8^ International Food Policy Research Institute Washington District of Columbia USA

**Keywords:** adolescent, anaemia, India, micronutrients, public health

## Abstract

Anaemia control programmes in India are hampered by a lack of representative evidence on anaemia prevalence, burden and associated factors for adolescents. The aim of this study was to: (1) describe the national and subnational prevalence, severity and burden of anaemia among Indian adolescents; (2) examine factors associated with anaemia at national and regional levels. Data (*n* = 14,673 individuals aged 10–19 years) were from India's Comprehensive National Nutrition Survey (CNNS, 2016–2018). CNNS used a multistage, stratified, probability proportion to size cluster sampling design. Prevalence was estimated using globally comparable age‐ and sex‐specific cutoffs, using survey weights for biomarker sample collection. Burden analysis used prevalence estimates and projected population from 2011 Census data. Multivariable logistic regression models were used to analyse factors (diet, micronutrient deficiencies, haemoglobinopathies, sociodemographic factors, environment) associated with anaemia. Anaemia was present in 40% of girls and 18% of boys, equivalent to 72 million adolescents in 2018, and varied by region (girls 29%–46%; boys 11%–28%) and state (girls 7%–62%; boys 4%–32%). Iron deficiency (ferritin < 15 μg/L) was the strongest predictor of anaemia (odds ratio [OR]: 4.68, 95% confidence interval [CI]: [3.21,6.83]), followed by haemoglobinopathies (HbA2 > 3.5% or any HbS) (OR: 2.81, 95% CI: [1.66,4.74]), vitamin A deficiency (serum retinol <20 ng/ml) (OR: 1.86, 95% CI: [1.23,2.80]) and zinc deficiency (serum zinc < 70 μg/L) (OR: 1.32, 95% CI: [1.02,1.72]). Regional models show heterogeneity in the strength of association between factors and anaemia by region. Adolescent anaemia control programmes in India should continue to address iron deficiency, strengthen strategies to identify haemoglobinopathies and other micronutrient deficiencies, and further explore geographic variation in associated factors.

## Key messages

- The Comprehensive National Nutrition Survey (CNNS) 2016–2018 is the only nationally representative survey to measure haemoglobin, haemoglobinopathies, biomarkers of micronutrient deficiencies, diet and social factors in Indian adolescents aged 10–19 years.
- Using CNNS data, we found that 28.5% of adolescents (girls: 39.6%, boys: 17.6%) were anaemic, with variation by region or state.
- Our prevalence estimates translate to 72 million adolescents being anaemic, with Uttar Pradesh having twice as many anaemic adolescents as any other state.
- Iron deficiency, haemoglobinopathies, vitamin A deficiency and zinc deficiency were associated with an increased risk of anaemia; these associations may vary subnationally.

## Graphical abstract

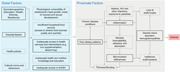

## 1 INTRODUCTION

Globally, one in four individuals aged 10–24 years (~430 million) suffer from anaemia, with the highest prevalence found in low‐ and middle‐income countries (Azzopardi et al., 2019). As the years between childhood and adulthood represent a sensitive period for developmental, physiological and behavioural changes, anaemia in this formative phase of life can reduce work capacity, impair neurocognitive and pubertal development and increase susceptibility to infections (Y. Balarajan et al., 2011; Haas & Brownlie, 2001; Scott et al., 2018). For adolescent girls also entering pregnancy (~21 million cases annually; Darroch et al., 2016), the consequences of anaemia extend to maternal and neonatal mortality as well as poor birth outcomes (Y. Balarajan et al., 2011; Black et al., 2013; Figueiredo et al., 2018; Haider et al., 2013).

India is home to 253 million adolescents 10–19 years of age, among the largest cohorts globally. Limited nationally representative nutrition survey data exist for this age group. National Family Health Surveys (NFHS) cover only the 15‐ to 19‐year age group and limited nutrition indicators. From 2005~2006 to 2019~2021, NFHS estimates indicate that anaemia prevalence among Indian adolescents aged 15~19 years has slightly increased (girls: 55.8% to 59.1%, boys: 30.2% to to 31.1%) (International Institute for Population Sciences, 2022).

There are nutritional and nonnutritional causes of anaemia: micronutrient deficiencies anind genetic blood disorders, including haemoglobinopathies, inflammation, infectious diseases and other physiological conditions such as menstruation and pregnancy (Y. Balarajan et al., 2011; Chaparro & Suchdev, 2019; GBD 2017 Disease and Injury Incidence and Prevalence Collaborators, 2018; Karakochuk et al., 2015; Nguyen et al., 2015; World Health Organization [WHO], 2017). In India, a recent analysis characterized types of anaemia among children and adolescents aged 1–19 years (Sarna et al., 2020). Among anaemic adolescents in this study, 21.3% had iron deficiency only (iron deficiency anaemia), 25.6% had folate or vitamin B12 deficiency without iron deficiency (folate or vitamin B12 deficiency anaemia), 18.2% had iron deficiency plus folate or vitamin B12 deficiency (dimorphic anaemia), 31.4% had no iron or folate or vitamin B12 deficiency (anaemia of other causes) and 3.4% had anaemia of inflammation. Anaemia is also associated with several individual‐ and household‐level characteristics such as education, age at marriage and wealth (Y. S. Balarajan et al., 2013; Chakrabarti et al., 2018; Nguyen et al., 2018; Prieto‐Patron et al., 2018). Existing studies on factors associated with anaemia in India mostly focus on women of reproductive age and children (Y. S. Balarajan et al., 2013; Nguyen et al., 2018; Varghese & Stein, 2019; Wirth et al., 2017), although there are some small studies on adolescents (Ahankari et al., 2017; Bharati et al., 2009; Mukherjee, 2016; Thomas et al., 2015; Toteja et al., 2006). Only one of these studies (Ahankari et al., 2017) assessed both nutritional and nonnutritional predictors of anaemia in a sample of 1010 adolescent girls living in a single district of Maharashtra. However, large nationally representative surveys investigating nutritional and nonnutritional correlates of anaemia in both boys and girls aged 10–19 years have not been available. Existing studies are limited in scope due to their small sample size, lack of representativeness and unavailability of comprehensive data on multiple risk factors of anaemia, particularly biological risk factors such as micronutrient deficiencies. To develop solutions for addressing anaemia in India, a country with large subnational variations in diets and living conditions, it is important to understand if factors associated with anaemia vary across geographies.

India's Adolescent Anaemia Control Programme was initiated as a pilot programme in 2000 in five states with three interventions targeting girls aged 10–19 years: weekly iron folic acid (IFA) supplementation, monthly nutrition and health education and biannual deworming prophylaxis (UNICEF, 2018). After a decade of evidence generation and phased implementation scale‐up, the Government of India universalized the programme and included boys as beneficiaries. In 2018, the programme added a ‘test and treat’ strategy with revised coating and dosage for the IFA supplements (aligned to WHO standards) and other selected interventions to tackle nonnutritional causes, rebranded as the ‘Anaemia‐Free India Programme’. Given the data gaps on prevalence and factors associated with adolescent anaemia, the Government of India conducted the Comprehensive National Nutrition Survey (CNNS) 2016–2018 (MoHFW et al., 2019). This is the first nationally representative survey in India to provide information on genetic, nutritional and nonnutritional factors implicated in the aetiology of anaemia for individuals below 20 years of age.

Using data from CNNS, this paper (1) describes the national and regional prevalence, severity and burden of anaemia in adolescents aged 10–19 years in India; and (2) examines factors associated with anaemia in this population, at both national and regional levels, to inform public health solutions to reduce adolescent anaemia in India.

## 2 METHODS

### 2.1 Data sources

**Figure 1:**
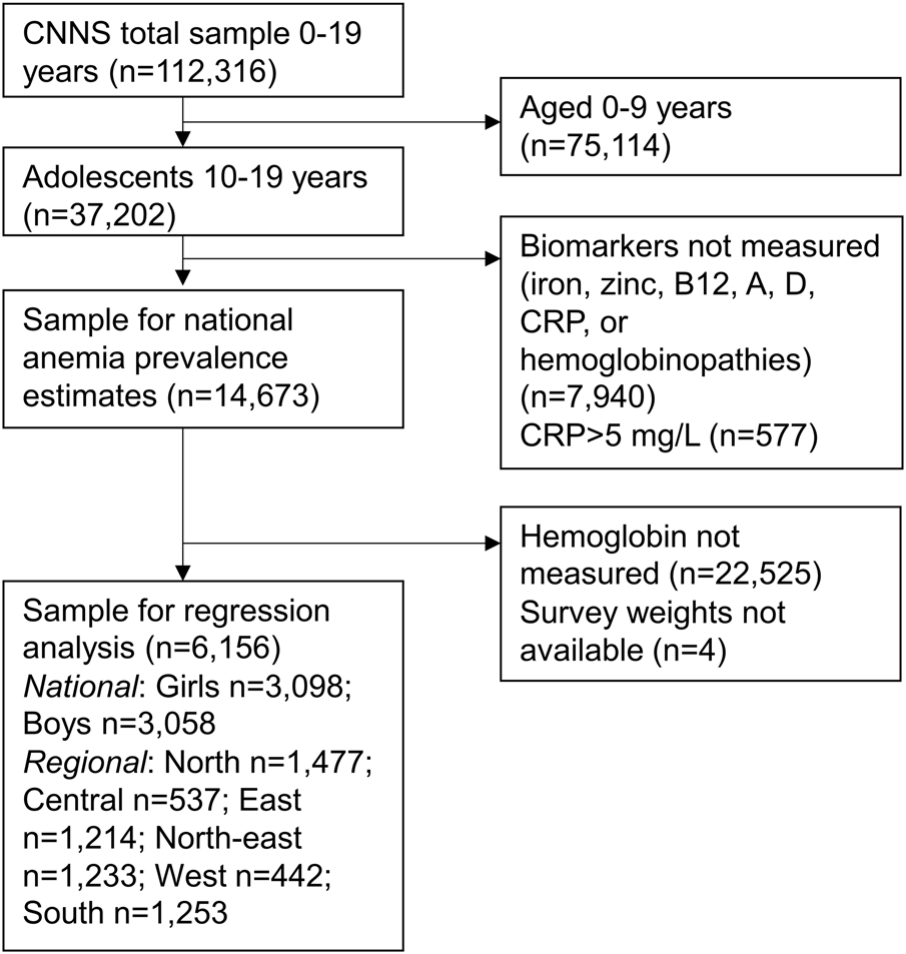
Sample flow diagram. CNNS, Comprehensive National Nutrition Survey; CRP, C‐reactive protein.

Data were from India's nationally representative CNNS, 2016–2018 conducted by the Population Council under the leadership of the MoHFW and UNICEF. International ethical approval was obtained from the Population Council's Institutional Review Board (IRB) in New York. National approval was obtained from the ethics committee of the Post Graduate Institute of Medical Education and Research in Chandigarh. For 10‐year‐old adolescents, informed consent was obtained from the parent/caregiver; for 11‐ to 17‐year‐old adolescents, informed consent was obtained from the parent/caregiver and adolescent; for 18‐ to 19‐year‐old adolescents, informed consent was obtained from the adolescent. Details on survey sampling procedures, data management and quality control for gold standard methods are published elsewhere (MoHFW et al., 2019). Briefly, the CNNS was designed to be representative of 28 states and 2 union territories, using a multistage, stratified, probability proportion to size cluster sampling methodology. Data were collected on household characteristics, environmental conditions, health status, dietary intake and anthropometry from 35,916 adolescents (10–19 years). For adolescents aged 10–14 years, a parent was asked to be present during the interview to help the adolescent respond. For all adolescents, parents answered questions about parental factors (e.g., education level) and household factors (e.g., sanitation). A subsample of 14,669 adolescents with a valid measure of haemoglobin (Hb) level and survey weights were included in the prevalence and burden analyses. Biological samples to assess micronutrient status were collected from two out of three adolescents, selected using systematic random sampling (MoHFW et al., 2019). Accounting for missing variables on biomarkers, the multivariable models included 6156 adolescents overall, of which 3058 boys and 3098 girls (Figure 1).

### 2.2 Biomarker measurement

The following anaemia‐related parameters were analysed: Hb, Hb variants (thalassaemia trait, HbA2 and sickle cell disease, HbS), serum ferritin, erythrocyte folate, serum levels of vitamin B12, retinol, zinc, 25‐hydroxyvitamin D (25(OH)D) and C‐reactive protein (CRP). Ten millilitres of blood were collected in the morning via antecubital venipuncture by trained phlebotomists, stored in trace element‐free vacutainers containing ethylenediaminetetraacetic acid‐K3 (Becton Dickinson) and transported from the field to the lab following rigorous quality control protocols (MoHFW et al., 2019). Blood samples were centrifuged at predesignated collection centres, aliquots were stored for laboratory analysis (whole blood at 5–7°C, serum frozen). Hb was estimated from whole blood using a haematology analyser (LH 750/780; Beckman Coulter). Hb variants were assessed by ion‐exchange high‐performance liquid chromatography (HPLC). Serum ferritin, erythrocyte folate, serum B_12_ and serum 25(OH)D were analysed by immunoassay using direct chemiluminescence. Serum zinc was estimated by flame atomic absorption spectroscopy. Serum retinol was analysed by HPLC reverse phase chromatography; CRP was measured using immunonephelometry. Standard quality control procedures were followed by all laboratories conducting biological analyses and laboratories participated in the US CDC VITAL‐EQA programme, which ships standard serum samples to participating laboratories and monitors the degree of variability and bias in assays (MoHFW et al., 2019).

### 2.3 Variables

#### 2.3.1 Outcome

Anaemia was the primary dependent variable, defined as altitude‐adjusted Hb < 11.5 g/dl for boys and girls 10–11 years, Hb < 12.0 g/dl for boys 12–14 years and girls 12–19 years and Hb < 13.0 for boys 15–19 years following standard WHO cutoffs (WHO, 2011a) (Supporting Information: Table S1). We used haemoglobin cutoffs currently recommended by the WHO as these cutoffs allow for international and interstudy comparability, although we note a recent call for a re‐examination of the cutoffs in the Indian population (Sachdev et al., 2021). For descriptive analyses, we also report the prevalence of mild (boys and girls 10–11 years: 11.0–11.4 g/dl; boys 12–14 years and girls 12–19 years: 11.0–11.9 g/dl; boys 15–19 years: 11.0–12.9 g/dl), moderate (8.0–10.9 g/dl) and severe (<8.0 g/dl) anaemia, again following standard WHO cutoffs (WHO, 2011a).

#### 2.3.2 Predictors

**Figure 2:**
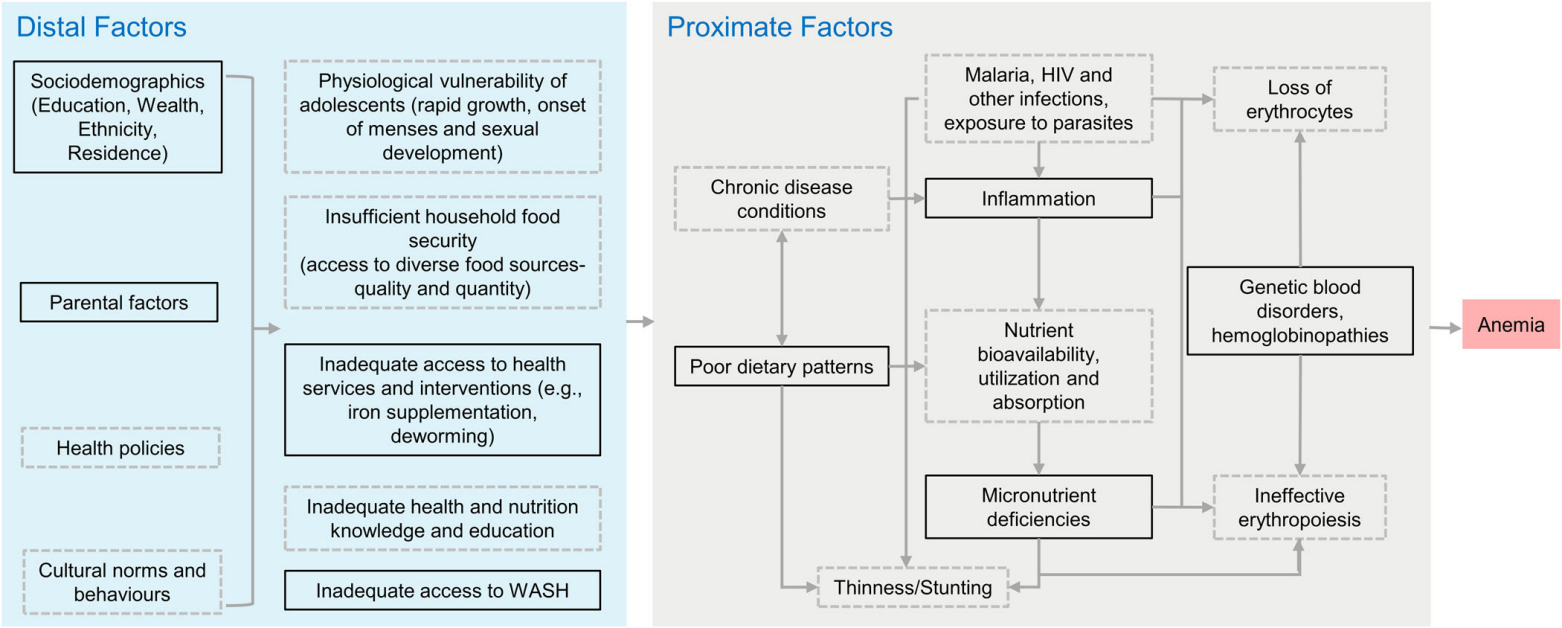
Conceptual framework for factors associated with anaemia in adolescents. Boxes with a solid outline are factors included in the regression analysis and boxes with a grey dotted outline are factors not included. Data on food security were collected in the Comprehensive National Nutrition Survey, but were not publicly available at the time of writing. WASH, water sanitation and hygiene.

We developed a conceptual framework based on existing frameworks (Y. Balarajan et al., 2011; Namaste et al., 2017), a review of the literature on risk factors and pathophysiology of anaemia (Chaparro & Suchdev, 2019), as well as data availability in the CNNS (Figure 2). Broadly, we classified factors (predictors) as either proximate or distal.

Proximate factors included dietary factors, micronutrient deficiencies and the presence of genes for haemoglobinopathies. Diet was assessed using a food frequency questionnaire, which asked adolescents how many days in a typical week they consumed various food groups. We constructed an ‘animal source foods’ indicator from eggs, fish, chicken or meat, coded as 1 if any of these foods were consumed at least one day per week and 0, if otherwise. Consumption of supplements or tablets included consumption of IFA supplements in the last week, multivitamin supplements in the last month and deworming tablets in the last 6 months. Micronutrient deficiencies were constructed as binary variables following sex‐ and age‐specific WHO cutoffs (Benoist, 2008; Namaste et al., 2017; WHO, 2011a, 2011b): iron deficiency as ferritin < 15 ng/ml; folate deficiency as erythrocyte folate < 151 ng/ml; vitamin B12 deficiency as serum B12 < 203 pg/ml; vitamin A deficiency as serum retinol < 20 μg/dl; vitamin D deficiency as serum 25(OH)D < 20 ng/ml; zinc deficiency as serum zinc < 66  g/dl to <74 μg/dl depending on sex, gender and fasting status (Supporting Information: Table S1). To account for the effects of inflammation on nutritional status biomarkers, individuals with CRP > 5 mg/L (3.9%) were excluded from the analyses (Namaste et al., 2017). An indicator of haemoglobinopathies was set equal to 1 if the adolescent had either thalassaemia trait (HbA2 > 3.5%) or sickle cell β thalassaemia (any HbS) and 0 if both tests were negative.

Distal factors included age (10–14 or 15–19 years), area of residence (rural/urban), wealth index, caste (scheduled caste/tribe, other backward classes (i.e., disadvantaged groups in India) and others), religion (Hindu and non‐Hindu), schooling status, parent's education (literate or illiterate), environmental factors (improved toilet, water and soap for handwashing), exposure to sources of information that may be educational with respect to health, such as mass media (newspaper/radio/TV), access to school meals) and geographical region of residence. Principal component analysis was used to construct the asset‐based household wealth index, following Demographic and Health Survey guidelines (Rustein & Johnson, 2004), which was further categorized into quintiles, where the highest quintile represented the richest and lowest quintile the poorest.

### 2.4 Statistical analysis

Descriptive analyses were conducted to report the characteristics of the study sample and provide regional estimates for anaemia (classified by types and severity). Maps were used to visualize state‐wise variability in anaemia prevalence and burden separately for boys and girls. Burden of anaemia was calculated as the product of the anaemia prevalence from CNNS data and the total eligible projected adolescents for each state in 2018 from Population Projections for India and States 2011–2036 (National Commission on Population, MoHFW, 2020), which estimated population on the basis of the Census 2011 age–sex data. National and regional multivariable logistic regression models were used to examine associations between anaemia and its associated factors among adolescent boys and girls. Model fit was assessed using the Hosmer–Lemshow goodness‐of‐fit test and Akaike and Bayesian information criterion (Vrieze, 2012). In the regional multivariable regression models, sex‐specific analyses were not conducted owing to limitations in sample size. To check for the robustness of the main results, we applied structural equation modelling with a maximum likelihood for estimation to account for missing values (Allison, 2003). All analyses accounted for the multistage cluster sampling design and survey weights specific to biomarker data. All analyses were conducted using Stata v.17.0.

## 3 RESULTS

### 3.1 Sample characteristics

Animal source foods were consumed at least once per week by 46.3% of adolescents and consumption was slightly more frequent in boys compared with girls (Table 1). Only 8.9% of adolescents consumed IFA supplements in the last week and 25.8% consumed deworming tablets in the last 6 months. Micronutrient deficiencies were common in both sexes, with iron and vitamin D deficiencies higher in girls and B12, folate and zinc deficiencies slightly higher in boys. Overall, folate deficiency (35.7%) was the most common and vitamin A deficiency was the least common (12.3%) micronutrient deficiency. Among distal factors, 74.3% of the sample lived in rural areas, 81.8% were Hindu, 32.7% were scheduled caste or tribe, and 77.3% were currently in school. Less than half of adolescents had parents who were both literate (42.4%) and around half had access to improved sanitation (55.8%) or materials for handwashing (50.9%). Sixty percent of adolescents were exposed to mass media, such as television, radio, newspaper or magazines less than once per week. One in four had access to a mid‐day meal in school.

**Table 1:** Sample characteristics of Indian adolescents aged 10–19 years by gender, 2016–2018

	Boys		Girls		Total	
	*N*	%	*N*	%	*N*	%
Proximate factors						
Dietary factors						
Consumed ASF (weekly)	7589	49.9	7084	42.6***	14,673	46.3
Consumed deworming tablets (last 6 months)	7589	24.5	7084	27.2	14,673	25.8
Consumed IFA supplements (last 1 week)	7589	7.3	7084	10.6***	14,673	8.9
Micronutrient deficiencies^a^						
Iron deficiency	5348	11.5	5197	31.3***	10,545	21.5
Vitamin B12 deficiency	5812	35.3	5570	26.6***	11,382	31.0
Folate deficiency	7056	38.5	6553	32.9***	13,609	35.7
Vitamin A deficiency	4345	11.6	4225	13.0	8570	12.3
Vitamin D deficiency	6460	13.8	6106	34.9***	12,566	24.2
Zinc deficiency	5881	34.7	5681	28.4***	11,562	31.5
Haemoglobinopathies	7156	5.0	6655	4.7	13,811	4.9
Distal factors						
Demographic characteristics						
Aged 10–14 years	7589	53.4	7084	51.7	14,673	52.6
Aged 15–19 years	7589	46.6	7084	48.3	14,673	47.4
Rural residence	7589	74.3	7084	74.3	14,673	74.3
Urban residence	7589	25.7	7084	25.7	14,673	25.7
Hindu religion	7589	83.0	7084	80.6	14,673	81.8
Caste/tribe						
Scheduled caste	7589	22.6	7084	22.3	14,673	22.4
Scheduled tribe	7589	11.1	7084	9.5	14,673	10.3
Other backward class	7589	42.0	7084	41.6	14,673	41.8
Other	7589	24.3	7084	26.7	14,673	25.5
Currently in school	7589	79.8	7084	74.8**	14,673	77.3
Wealth index						
Poorest	7589	18.3	7084	17.7	14,673	18.0
Poor	7589	21.1	7084	20.3	14,673	20.7
Middle	7589	20.2	7084	21.1	14,673	20.6
Rich	7589	19.9	7084	22.6	14,673	21.2
Richest	7589	20.5	7084	18.3	14,673	19.4
Parent's education						
Both illiterate	7589	23.1	7084	23.4	14,673	23.3
Either literate	7589	33.6	7084	35.0	14,673	34.3
Both literate	7589	43.2	7084	41.5	14,673	42.4
Environmental factors						
Hygiene and sanitation						
Access to improved sanitation^b^	7589	55.8	7084	55.8	14,673	55.8
Access to soap and water for handwashing	7589	50.4	7084	51.4	14,673	50.9
Mass media exposure level^c^						
Low	7589	56.3	7084	62.7**	14,673	59.5
Medium	7589	31.8	7084	28.8	14,673	30.3
High	7589	11.9	7084	8.5	14,673	10.2
Access to school‐based services						
Received mid‐day meal	7589	25.2	7084	24.8	14,673	25.0

*Note*: *N* column is the denominator.

Abbreviations: ASF, animal source foods; IFA, iron folic acid.

^a^ Definitions were as follows: iron deficiency (serum ferritin < 15 ng/ml), vitamin B12 deficiency (serum B12 < 203 pg/ml), folate deficiency (erythrocyte folate < 151 ng/ml), vitamin A deficiency (serum retinol < 20 μg/ml), vitamin D deficiency (serum 25(OH)D < 20 ng/ml), zinc deficiency (nonpregnant females: <70 μg/dl [morning fasting], <66 μg/dl [morning nonfasting]; males: <74 μg/dl [morning fasting], <70 μg/dl [morning nonfasting]).

^b^ Improved sanitation includes flush or pour toilet, piped sewer system, septic tank, pit latrine, and VIP toilet, pit latrine with slab and composting toilets.

^c^ Mass media includes watching television, listening to the radio and reading newspaper/magazine. Low level was defined as exposure less than once a week, medium as at least once a week and high level as almost every day.

** *p* < 0.01

*** *p* < 0.001.

### 3.2 Prevalence of adolescent anaemia at national, regional and state levels

**Figure 3:**
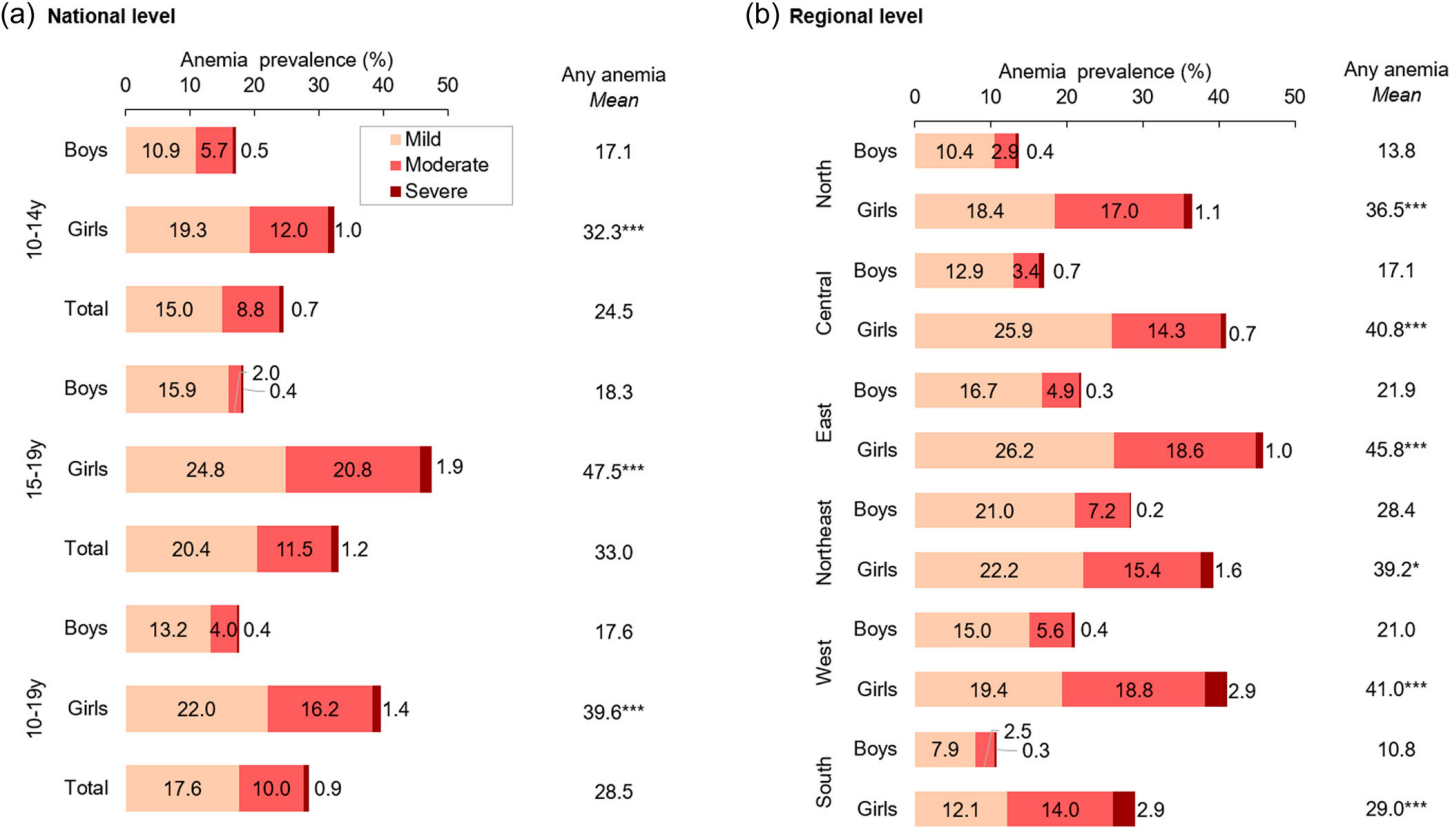
Prevalence and severity of anaemia among Indian adolescents, 2016–2018. Panel (a) shows prevalence at the national level by age group and sex. Panel (b) shows prevalence for all ages (10–19 years) by region and sex. The ‘Any anaemia’ column to the right of each panel shows the total prevalence of anaemia (severity categories combined). Severity categories are defined according to standard age‐ and gender‐specific World Health Organization cutoffs: 10–11 years: <11.5 g/dl (mild: 11.0–11.4; moderate: 8.0–10.9; severe: <8.0); 12–14 years: <12 g/dl (mild: 11.0–11.9; moderate: 8.0–10.9; severe: <8.0); 15–19 years males: <13 g/dl (mild: 11.0–12.9; moderate: 8.0–10.9; severe: <8.0]; 15–19 years females: <12 g/dl (mild: 11.0–11.9; moderate: 8.0–10.9; severe: <8.0). See Supporting Information: Table S2 for states included within each region.

**Figure 4:**
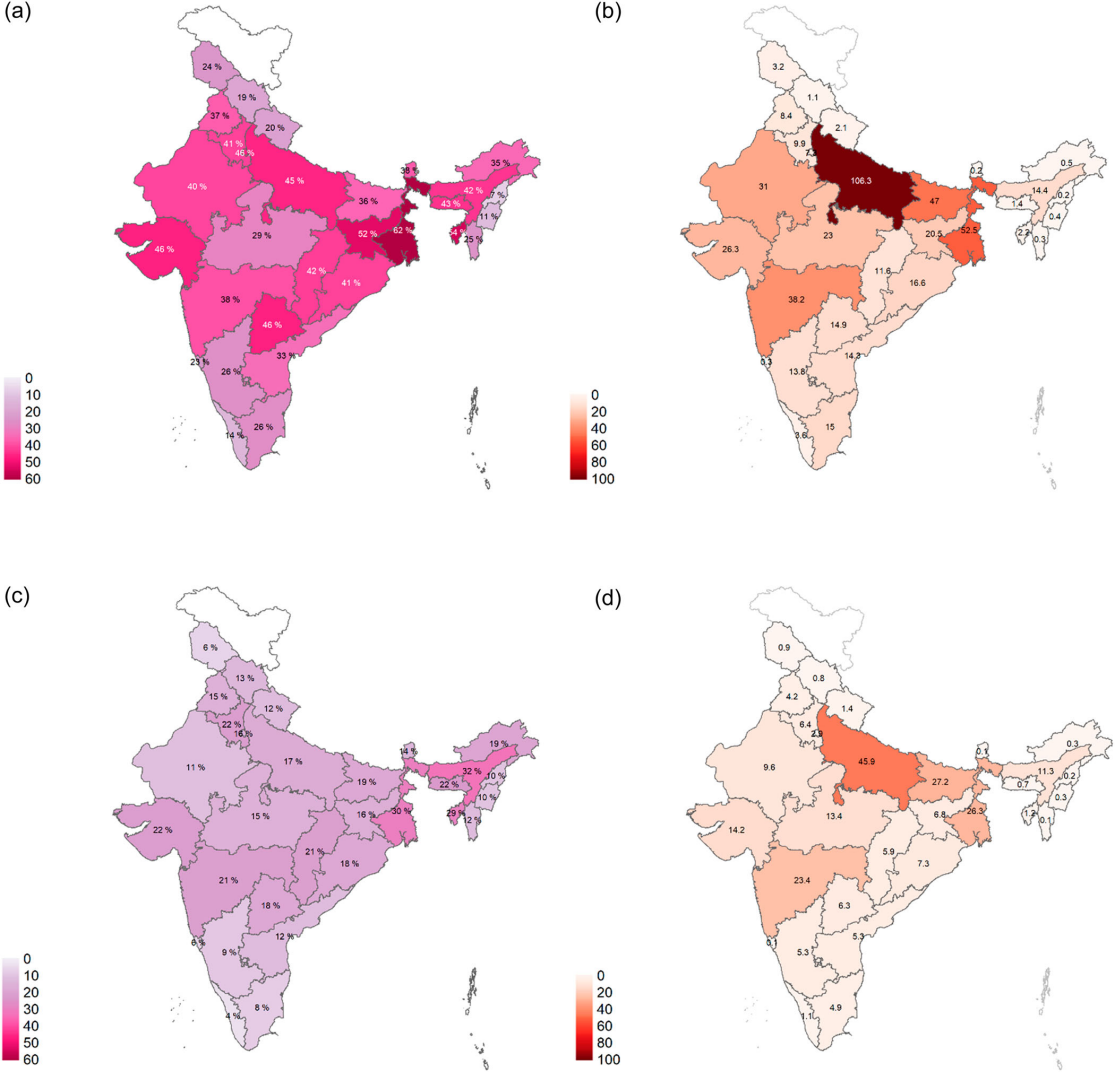
Prevalence and burden of anaemia among Indian adolescent girls and boys aged 10–19 years by state, 2016–2018. Prevalence categories are defined according to WHO public‐health significance cut‐offs (see Supporting Information: Table S1). Burden numbers are in thousands (a) Girls aged 10–19 years, % anaemic, (b) girls aged 10–19 years, number anaemic (thousands), (c) boys aged 10–19 years, % anaemic and (d) boys aged 10–19 years, number anaemic (thousands).

The prevalence of anaemia among adolescents was 28.5% overall, corresponding to more than 72 million adolescents (Figure 3, panel a). Anaemia was higher in girls (39.6%, 48.7 million) than boys (17.6%, 23.7 million). In terms of severity, 17.6% of adolescents had mild anaemia, 10.0% had moderate anaemia and 0.9% had severe anaemia. Anaemia was highest among girls aged 15–19 years (47.5%) and lowest among adolescent boys aged 10–14 years (17.1%). Anaemia prevalence varied widely across regions, ranging from 29.0% in the South to 45.8% in the East for girls; and from 10.8% in the South to 28.4% in the Northeast for boys (Figure 3, panel b). Among girls, 13 states had ≥40% prevalence of anaemia, with the highest prevalence in West Bengal (62.0%) (Figure 4 and Supporting Information: Table S2). Assam had the highest prevalence of anaemia in boys (32.0%), while Kerala had the lowest (4.1%). Uttar Pradesh carried the largest burden of anaemia with 10.6 million girls and 4.6 million boys suffering from anaemia.

### 3.3 Factors associated with anaemia in adolescents

#### 3.3.1 National models

Among proximate factors, dietary factors were not associated with anaemia in the overall model but consuming ASF at least weekly among girls (odds ratio [OR]: 1.52, 95% confidence interval [CI]: [1.04, 2.23]) and having consumed IFA in the past week among boys (OR: 1.92, 95% CI: [1.12,3.29]) were predictive of increased odds of anaemia (Table 2). Several micronutrient deficiencies were associated with anaemia. In the overall model, iron deficiency (OR: 4.68, 95% CI: [3.21,6.82]), vitamin A deficiency (OR: 1.86, 95% CI: [1.23,2.80]) and zinc deficiency (OR: 1.32, 95% CI: [1.02,1.72]) were positively associated with anaemia while folate deficiency (OR: 0.60, 95% CI: [0.46,0.80]) was negatively associated with anaemia. Vitamin B12 and D deficiencies were not associated with anaemia. The presence of genes for thalassaemia trait or sickle‐cell β‐thalassaemia predicted higher anaemia in both sexes (OR: 2.81, 95% CI: [1.66,4.74]).

**Table 2:** Odds of anaemia by proximate and distal factors in Indian adolescents aged 10–19 years, results from the national‐level multivariable logistic regression models

	Boys (*n* = 3058)		Girls (*n* = 3098)		Overall (*n* = 6156)	
	OR	[95% CI]	OR	[95% CI]	OR	[95% CI]
Proximate factors						
Dietary factors						
Consumed ASF (weekly)	0.86	[0.56,1.30]	1.52*	[1.04,2.23]	1.16	[0.87,1.56]
Consumed deworming tab (last 6 months)	1.13	[0.74,1.71]	1.10	[0.78,1.54]	1.11	[0.84,1.46]
Consumed IFA supplements (last 1 week)	1.92*	[1.12,3.29]	0.95	[0.59,1.54]	1.18	[0.80,1.72]
Micronutrient deficiencies						
Iron deficiency	5.72***	[3.46,9.45]	4.65***	[2.98,7.25]	4.68***	[3.21,6.83]
Vitamin B12 deficiency	0.86	[0.53,1.39]	1.05	[0.65,1.71]	0.99	[0.68,1.43]
Folate deficiency	0.59*	[0.36,0.98]	0.57**	[0.41,0.81]	0.60***	[0.46,0.80]
Vitamin A deficiency	2.16*	[1.20,3.88]	1.55	[0.92,2.60]	1.86**	[1.23,2.80]
Vitamin D deficiency	1.01	[0.66,1.54]	0.91	[0.61,1.36]	0.95	[0.70,1.28]
Zinc deficiency	1.46	[0.98,2.17]	1.27	[0.90,1.78]	1.32*	[1.02,1.72]
Haemoglobinopathies	2.91**	[1.32,6.42]	3.04**	[1.45,6.37]	2.81***	[1.66,4.74]
Distal factors						
Sociodemographic factors						
Aged 15–19 years (ref: 10–14 years)	1.90*	[1.15,3.14]	1.55	[1.00,2.40]	1.57*	[1.10,2.25]
Female					2.56***	[1.94,3.39]
Currently in school	0.65	[0.34,1.23]	0.98	[0.59,1.62]	0.87	[0.58,1.30]
Wealth Index (ref: richest)						
Poorest	0.65	[0.23,1.84]	1.08	[0.43,2.72]	0.91	[0.43,1.91]
Poor	0.65	[0.26,1.61]	1.52	[0.73,3.15]	1.18	[0.66,2.11]
Middle	0.78	[0.39,1.59]	1.40	[0.79,2.47]	1.11	[0.71,1.73]
Rich	0.73	[0.39,1.39]	1.49	[0.83,2.70]	1.22	[0.76,1.95]
Either parent illiterate (ref: both literate)	1.56	[0.97,2.52]	0.93	[0.62,1.40]	1.13	[0.82,1.54]
Environmental factors						
Access to improved sanitation	0.66	[0.42,1.04]	1.18	[0.77,1.80]	0.95	[0.69,1.32]
Access to soap and water for handwash	1.07	[0.68,1.68]	0.83	[0.54,1.27]	0.92	[0.65,1.28]
Mass media exposure level (ref: high)						
Low	1.16	[0.59,2.27]	0.92	[0.52,1.62]	1.04	[0.67,1.61]
Medium	0.67	[0.34,1.31]	1.14	[0.62,2.09]	1.02	[0.63,1.66]
Received mid‐day meal in school	1.44	[0.79,2.63]	0.67	[0.42,1.06]	0.88	[0.60,1.29]

*Note*: Separate models were run for boys, girls and overall (sexes combined). All models controlled for residence (rural/urban), religion, caste and region.

Abbreviations: ASF, animal source foods; IFA, iron folic acid; ref, reference category.

* *p* < 0.05

** *p* < 0.01

*** *p* < 0.001 from logistic multivariable regression models.

Among distal factors, adolescents aged 15–19 years had higher odds of anaemia (OR: 1.57, 95% CI: [1.10,2.25]) compared with those aged 10–14 years and girls were more likely to be anaemic than boys (OR: 2.56, 95% CI: [1.94,3.39]). Being in school, wealth, parental literacy, sanitation, hygiene, mass media exposure and access to school meals were not significantly associated with anaemia. Sensitivity analysis using structural equation models which accounted for missing values with biomarkers yielded similar findings (results not shown).

#### 3.3.2 Regional models

Repeating our multivariable regression analyses by region (girls and boys combined) revealed some regional heterogeneity in relationships (Table 3). Dietary factors were generally not associated with anaemia, except for consuming IFA in the Northeast (OR: 3.43, 95% CI: [1.04,11.34]) and deworming tablets in the South (OR: 1.77, 95% CI: [1.04,3.02]). Iron deficiency was strongly associated with anaemia in all regions except the Central region, with ORs ranging from 4.42 in the Northeast to 28.13 in the West. Vitamin B12 deficiency was negatively associated with anaemia in the West (OR: 0.17, 95% CI: [0.05,0.52]), but not in other regions. The odds of anaemia were also lower in adolescents with folate deficiency in the East (OR: 0.57, 95% CI: [0.34, 0.97]) and South (OR: 0.40, 95% CI: [0.24,0.67]). Vitamin A and D deficiencies were not associated with anaemia in any region. Zinc deficiency predicted higher odds of anaemia in the North (OR: 1.61, 95% CI: [1.06,2.43]) and South (OR: 2.55, 95% CI: [1.57,4.14]). Haemoglobinopathies were a strong positive predictor of anaemia in the North, Northeast and South. Among distal factors, older age (15–19 years) and female sex were associated with a higher likelihood of anaemia in most regions. Being in school and parental literacy was not associated with anaemia in any region. In the South and West regions, adolescents from lower wealth quintiles had higher odds of anaemia. Sanitation and hygiene were also not associated with anaemia except for improved sanitation in the Northeast, although in the opposite direction as expected (OR: 3.58, 95% CI: [1.18,10.80]). Being exposed to mass media and receiving a mid‐day meal in school was not associated with anaemia.

**Table 3:** Odds of anaemia by proximate and distal factors in Indian adolescents aged 10–19 years, results from regional‐level multivariable logistic regression models

	North (*n* = 1477)		Central (*n* = 537)		East (*n* =1214)		Northeast (*n* = 1223)		West (*n* =442)		South (*n* = 1255)	
	OR	[95% CI]	OR	[95% CI]	OR	[95% CI]	OR	[95% CI]	OR	[95% CI]	OR	[95% CI]
Proximate factors												
Dietary factors												
Consumed ASF (weekly)	1.18	[0.76,1.86]	1.23	[0.64,2.36]	1.30	[0.84,2.00]	2.27	[0.87,5.91]	0.36	[0.12,1.04]	0.89	[0.50,1.56]
Consumed deworming tab (last 6 months)	0.66	[0.41,1.06]	1.51	[0.66,3.44]	1.04	[0.66,1.63]	0.69	[0.29,1.68]	1.50	[0.37,6.13]	1.77*	[1.04,3.02]
Consumed IFA supplements (last 1 week)	1.13	[0.54,2.39]	0.54	[0.14,2.10]	1.71	[0.94,3.08]	3.43*	[1.04,11.34]	3.66	[0.68,19.78]	0.60	[0.24,1.46]
Micronutrient deficiencies												
Iron deficiency	6.71***	[4.40,10.24]	1.75	[0.81,3.81]	7.72***	[4.35,13.69]	4.42**	[1.45,13.43]	28.13***	[8.77,90.19]	11.67***	[7.08,19.23]
Vitamin B12 deficiency	1.31	[0.83,2.08]	0.77	[0.41,1.46]	1.38	[0.77,2.47]	0.56	[0.11,2.82]	0.17**	[0.05,0.52]	1.06	[0.61,1.83]
Folate deficiency	1.16	[0.62,2.16]	0.99	[0.46,2.11]	0.57*	[0.34,0.97]	0.45	[0.19,1.07]	0.56	[0.23,1.39]	0.40***	[0.24,0.67]
Vitamin A deficiency	1.76	[0.82,3.75]	1.81	[0.86,3.79]	1.20	[0.58,2.51]	2.55	[0.63,10.38]	0.61	[0.11,3.25]	1.51	[0.84,2.73]
Vitamin D deficiency	0.83	[0.49,1.39]	0.96	[0.47,1.95]	1.09	[0.69,1.74]	1.37	[0.53,3.51]	0.51	[0.18,1.50]	0.81	[0.47,1.41]
Zinc deficiency	1.61*	[1.06,2.43]	0.92	[0.48,1.78]	1.22	[0.81,1.86]	1.12	[0.49,2.56]	1.65	[0.60,4.54]	2.55***	[1.57,4.14]
Haemoglobinopathies	15.38***	[5.04,46.92]	1.80	[0.66,4.91]	2.22	[0.96,5.15]	27.31**	[3.40,219.44]	7.96**	[1.88,33.69]	3.50	[0.52,23.67]
Distal factors												
Sociodemographic factors												
Aged 15–19 years (ref: 10–14 years)	3.37***	[2.00,5.68]	1.02	[0.50,2.06]	1.37	[0.86,2.18]	2.02	[0.78,5.27]	2.09	[0.72,6.10]	3.52***	[1.94,6.38]
Female	3.14***	[1.99,4.95]	3.48***	[1.72,7.05]	2.37***	[1.52,3.67]	1.05	[0.39,2.84]	2.39	[0.96,5.98]	2.06**	[1.23,3.45]
Currently in school	1.99	[0.91,4.38]	0.67	[0.32,1.40]	0.74	[0.42,1.29]	0.44	[0.12,1.57]	1.02	[0.25,4.15]	0.97	[0.30,3.20]
Wealth Iindex (ref: richest)												
Poorest	0.22	[0.02,2.28]	0.24	[0.05,1.03]	1.41	[0.52,3.85]	3.02	[0.65,14.06]	21.85	[0.99,482.82]	7.26*	[1.16,45.31]
Poor	2.01	[0.49,8.28]	0.41	[0.11,1.57]	1.26	[0.52,3.08]	1.25	[0.26,5.96]	43.63**	[3.13,607.53]	3.09*	[1.26,7.62]
Middle	1.19	[0.54,2.59]	0.51	[0.16,1.60]	1.02	[0.50,2.10]	1.39	[0.48,4.06]	14.57*	[1.83,115.79]	2.15	[0.97,4.75]
Rich	1.12	[0.67,1.86]	0.79	[0.23,2.75]	1.26	[0.66,2.42]	0.50	[0.16,1.50]	5.28**	[1.65,16.93]	1.42	[0.76,2.66]
Either parent illiterate (ref: both literate)	0.89	[0.53,1.49]	1.21	[0.60,2.44]	1.00	[0.63,1.60]	0.60	[0.20,1.75]	0.56	[0.10,3.16]	1.35	[0.81,2.24]
Environmental factors												
Access to improved sanitation	0.53	[0.23,1.22]	0.76	[0.36,1.58]	0.93	[0.54,1.59]	3.58*	[1.18,10.80]	1.14	[0.30,4.31]	1.75	[1.00,3.07]
Access to soap and water for handwash	1.08	[0.61,1.92]	1.11	[0.60,2.08]	0.72	[0.46,1.13]	0.78	[0.33,1.81]	1.76	[0.46,6.67]	1.20	[0.73,1.98]
Mass media exposure level (ref: high)												
Low	2.10	[0.88,5.05]	0.59	[0.23,1.50]	1.91	[0.94,3.87]	1.25	[0.33,4.72]	4.70	[0.61,36.24]	0.88	[0.39,1.97]
Medium	1.34	[0.56,3.21]	0.64	[0.23,1.76]	1.32	[0.61,2.85]	0.70	[0.17,2.86]	6.73	[0.82,55.48]	1.49	[0.66,3.35]
Received mid‐day meal in school	1.56	[0.78,3.14]	0.57	[0.21,1.55]	0.91	[0.53,1.56]	0.70	[0.23,2.10]	0.54	[0.13,2.22]	1.28	[0.40,4.10]

*Note*: All models controlled for residence, religion and caste. For a list of states in each region, see Supporting Information: Table S2.

Abbreviations: ASF, animal source foods; IFA, iron folic acid; ref, reference category.

* *p* < 0.05

** *p* < 0.01

*** *p* < 0.001 from multivariable logistic regression models for each region.

## 4 DISCUSSION

### 4.1 Summary of findings

Using data from the largest nutrition survey in Asia, our study reports on the prevalence and factors associated with anaemia in Indian adolescents aged 10–19 years at both national and subnational levels. Overall anaemia prevalence in this age group was 28.5%, indicating a moderate public health problem according to cutoffs currently recommended by the WHO (2011a). However, among girls, anaemia was found to be a severe public health problem (prevalence ≥ 40%) in 13 states. In absolute terms, anaemia affected more than 72 million Indian adolescents, with females accounting for about two‐thirds of the burden. Our regression analyses show that while iron deficiency showed the strongest association with anaemia, other factors such as vitamin A and zinc deficiencies, haemoglobinopathies, older age and household wealth were also associated with anaemia prevalence in this population, with variation in the associations by region.

### 4.2 Comparison with other studies

Other studies have reported on the strong association between iron deficiency and anaemia in Indian adolescent boys and girls, but none to our knowledge have done so at national and regional levels. Our findings are consistent with recent studies on school‐aged children and adolescents from Nepal (Ford et al., 2020a), Kuwait (Shaban et al., 2020) and Bangladesh (Ahmed et al., 2000), and community‐based studies on adolescent girls in India (Patel et al., 2017; Srivastava et al., 2016; Thomas et al., 2015). Similar associations between anaemia and iron deficiency are well documented in women of reproductive age and children (Engle‐Stone et al., 2017; Ford et al., 2020b; George et al., 2012; Petry et al., 2019; Wirth et al., 2017). Although IFA has been recommended and guidelines for weekly IFA supplementation exist, coverage of IFA supplements among adolescents was low (9%), suggesting supply, access and/or adherence issues. Consumption of IFA supplements in the last week was not a significant predictor of anaemia in the overall national model or in any region, except for among boys where IFA consumption predicted higher odds of anaemia. We speculate that this may simply reflect better adherence among anaemic boys compared with nonanaemic boys.

Our findings on associations between vitamin A deficiency and anaemia are aligned with previous studies on adolescents in Nepal (Ford et al., 2020a) and Bangladesh (Ahmed et al., 2000). Vitamin A deficiency is known to be associated with reduced iron binding capacity and transferrin saturation. Additionally, vitamin A also modulates iron homeostasis by regulating hepcidin synthesis and plays a critical role in immune modulation, with vitamin A deficiency increasing susceptibility to anaemia of infection (da Cunha et al., 2014; Semba & Bloem, 2002). Similar findings on the relationship between zinc status and anaemia have been reported from studies on school‐aged children and adolescents in New Zealand (Houghton et al., 2016) and Turkey (Atasoy & Bugdayci, 2018). Folate deficiency was inversely associated with anaemia in our study. Previous cross‐sectional studies have also reported similar findings (Arsenault et al., 2009; Caicedo et al., 2010; Morris et al., 2007; Rogers et al., 2003; Saraya et al., 1973). Mechanistically, this inverse association has been attributed to competition between iron and folate. The intestinal transporter protein, essential for normal iron and folate absorption and homeostasis (PCFT/HCP1), has a higher affinity for folate (Arsenault et al., 2009; Qiu et al., 2006; Shayeghi et al., 2005). This can lead to a competitive reduction of haem‐–iron absorption, resulting in lower haemoglobin synthesis (Arsenault et al., 2009). In contrast, an analysis of nationally representative data from 10 surveys (Engle‐Stone et al., 2017; Merrill et al., 2017) studying factors associated with anaemia in children and women of reproductive age showed no association between anaemia and folate deficiency, a finding additionally supported by a systematic review on the haematologic effects of folate deficiency (Metz, 2008). Therefore, we suggest caution when interpreting our finding on folate deficiency being associated with reduced odds of anaemia.

The presence of thalassaemia trait (HbE) or sickle‐cell β‐thalassaemia was associated with higher odds of anaemia. Genetic haemoglobin disorders can be homozygous (manifesting in the disease) or heterozygous (trait) and can lead to defective formation of haemoglobin, thereby increasing the risk of anaemia. In India, previous studies have investigated the prevalence of thalassaemia traits and sickle cell disease (Bhukhanvala et al., 2012; Madan et al., 2010; Mohanty et al., 2008, 2014); however, these studies did not examine the association of haemoglobinopathies with anaemia in adolescents. Thalassaemia traits have been found to be associated with anaemia in a nationally representative sample of Malawian children (McGann et al., 2018), cohorts of rural children in Karnataka (Pasricha et al., 2010), India and young children and women from Cambodia (George et al., 2012; Karakochuk et al., 2015). This finding underscores the importance of screening programmes in schools for the assessment of thalassaemia traits, supplemented by necessary counselling. India currently has detailed guidance from the National Health Mission on population‐level screening programmes for the detection of carriers of β‐thalassaemia HbS and HbE (MoHFW, 2016; Patra et al., 2015). The policy on prevention and control of haemoglobinopathies also recommends community education and awareness generation, though the status of implementation for these programmes remains unclear.

We found few associations between nonnutritional or distal factors and anaemia other than being older and female being associated with higher odds of anaemia compared with being younger and male. Wealth, parental literacy, sanitation, hygiene, mass media exposure and receiving free school meals were not associated with anaemia. Previous studies that have found associations between nonnutritional factors and anaemia have not controlled for micronutrient status (Ahankari et al., 2017; Nguyen et al., 2018). It may be that inclusion of additional proximate factors masks associations between distal factors and anaemia. To test this hypothesis, we ran additional exploratory models (Supporting Information: Table S2). In bivariate analysis, odds of anaemia were lower among adolescents in school (compared with those out of school), from richer households, when parents were literate, when adolescents had access to soap and water for handwashing, when media exposure was high and when a mid‐day meal was received in school. All of these associations became nonsignificant in the multivariable models. In contrast, bivariate associations between proximate factors and anaemia remained significant when all proximate factors were included in a multivariable model, and in the full model with distal factors added. It is not surprising to us that biological factors are more strongly associated with a biological outcome compared with nonbiological factors as biological factors are further along the pathway to the outcome (Figure 2). Interventions that target distal factors are likely to operate through proximate pathways to eventually affect the outcome. For example, a sanitation intervention would be expected to reduce disease and gut inflammation, increasing absorption of micronutrients, thus resolving micronutrient deficiencies that were directly causing anaemia.

### 4.3 Strengths and limitations

Our study provides a current description of the prevalence, burden and associated factors of anaemia in a nationally representative sample of Indian adolescents. In addition to providing data on an understudied age group—NFHS includes 15– 19‐year‐old but not 10–14‐year‐old adolescents—CNNS uses gold standard methods for estimation of anaemia and other micronutrient deficiencies. Most previous field surveys, including NFHS, have used HemoCue 201+ for estimating haemoglobin concentrations from capillary blood samples, which is known to overestimate anaemia prevalence in hot and humid environments (Whitehead et al., 2019). Inaccuracies with the HemoCue method have also been attributed to differential dilution pressure due to milking, skin temperature and depth of needle penetration (Boghani et al., 2017; Gwetu et al., 2013). CNNS used an automated haematology analyser, which provides higher precision and accuracy and is based on the WHO‐recommended cyanohaemoglobin method of estimation (Abraham et al., 2020; MoHFW et al., 2019). The different methods of Hb assessment between NFHS and CNNS are responsible for the different prevalence estimates.

Our study is not without methodological limitations. First, CNNS is cross‐sectional in nature and hence precludes inference of any causal relationship between anaemia and its associated factors. Second, exclusion of individuals with elevated CRP may introduce bias, but as AGP was not available and as most CRP values were clustered at low CRP levels, it was a challenge to address the effects of inflammation on acute‐phase proteins using a regression‐based approach (Namaste et al., 2017). However, we conducted a sensitivity analysis to compare the results of regression models with and without individuals with elevated CRP and found no differences (Supporting Information: Table S2). Third, dietary data only included the frequency of food group consumption; a detailed dietary assessment is necessary to study the potential contribution of dietary constituents to adolescent anaemia. Fourth, our regional analysis should be treated as exploratory and interpreted with caution due to the limited sample size, particularly in the Central (*n* = 537) and West (*n* = 442) regions. This issue may underlie unexpected significant associations such as the negative association between vitamin B12 deficiency and anaemia in the West. Ideally, we would have been able to conduct a state‐level regression analysis given that many decisions are made at the state level in India, but the sample with biomarker data was far too small for such an analysis.

### 4.4 Policy and programme context and implications

India is among the few countries in South Asia and the world to have a comprehensive programme to address anaemia in the adolescent population. The *Anaemia Free India* programme (MoHFW, Government of India, 2018), launched by the Government of India in 2018 and which targets both boys and girls, adopts a holistic approach combining weekly IFA supplementation, biannual deworming, nutrition and health education, fortification, annual checkups, anaemia test, treat and talk camps. The programme also strengthens existing programmes that address nonnutritional causes of anaemia (bed nets in malaria‐endemic areas, sickle cell anaemia, fluorosis). Beyond the *Anaemia Free India* programme, additional complementary interventions that may be particularly important for adolescents include the provision of mid‐day meals (which, currently, are not offered to adolescents 15 years or older under India's Mid‐Day Meal Scheme, PM‐POSHAN), cash transfers to keep girls in school and prevent early marriage and special programmes targeted at regions with a high prevalence of sickle cell anaemia (Aguayo et al., 2013; MoHFW, Government of India, 2012; Sethi et al., 2017). Anaemia camps to test, treat and counsel adolescents, which are a part of the *Anaemia Free India* programme, should target vulnerable populations— especially adolescent girls out of school—engage with school health programmes, and screen for haemoglobinopathies.

While there is a growing consensus that nutrition‐specific strategies alone will not end anaemia, our findings highlight the need for nutrition‐specific strategies that focus on iron but also address deficiencies in other micronutrients such as vitamin A and zinc. The IFA supplementation strategy has supply and compliance barriers (Ramakrishnan et al., 2012; Sethi et al., 2017), which still need to be addressed. Evidence on the effectiveness of anaemia reduction in adolescents using multiple micronutrient supplementation is limited and more research, particularly large‐scale implementation and operational research, is needed in this direction. Indian diets are primarily cereal‐based and vegetarian, and systematic and continuous investments in large‐scale communication campaigns on improving healthy diets through locally available and affordable foods rich in micronutrients are needed. As India's food security schemes scale‐up fortification of staple food items such as rice, wheat and oil with safe levels of micronutrients, parallel efforts are needed to promote healthy diets. Recently, the government has launched a large‐scale effort to distribute fortified rice through its safety nets, and such efforts should be evaluated to better understand their effectiveness in terms of reducing micronutrient deficiencies and anaemia (Department of Food and Public Distribution, 2020). Overall anaemia reduction requires a mix of strategies, which are already outlined in *Anaemia Free India*. This programme requires nested evaluations in select geographies where all interventions are codelivered with adequate coverage. Given some heterogeneity in factors associated with anaemia across regions and the existing differentials between states in terms of progress in programme implementation, context‐specific programming can be considered.

CNNS is a major step forward in understanding the nutritional status of the young Indian population. However, anaemia prevalence increased in many states from 2015–2016 to 2019–2021 (MoHFW, Government of India, 2019), thus a deeper understanding of how to reverse the trend is urgently needed. Our findings, which align with a recent call for interventions and policies to cut across sectors to improve adolescent nutrition (Hargreaves et al., 2021), suggest that addressing iron deficiency is a logical starting point, but strategies beyond IFA supplementation are needed to tackle the persistent and prevalent problem of adolescent anaemia in India.

## AUTHOR CONTRIBUTIONS

Anwesha Lahiri, Phuong Hong Nguyen and Samuel Scott analysed the data. Anwesha Lahiri, Samuel Scott and Vani Sethi wrote the paper. Arjan de Wagt, Purnima Menon, Kapil Yadav, Mini Varghese, William Joe and Sheila C. Vir provided critical interpretation and inputs on the paper. Samuel Scott had responsibility for the final content. All authors read and approved the final manuscript.

## CONFLICT OF INTEREST

The authors declare no conflict of interest.

## Supporting information

Supporting information.

## Data Availability

The data that support the findings of this study are available from the corresponding author upon reasonable request.

## References

[mcn13391-bib-0001] Abraham, R. A. , Agrawal, P. K. , Johnston, R. , Ramesh, S. , Porwal, A. , Sarna, A. , Acharya, R. , Khan, N. , Sachdev, H. S. , Kapil, U. , Saxena, R. , Janmohamed, A. , Wagt, A. , Deb, S. , Khera, A. , & Ramakrishnan, L. (2020). Comparison of hemoglobin concentrations measured by HemoCue and a hematology analyzer in Indian children and adolescents 1–19 years of age. International Journal of Laboratory Hematology, 42, e155–e159. 32301247 10.1111/ijlh.13209

[mcn13391-bib-0002] Aguayo, V. M. , Paintal, K. , & Singh, G. (2013). The Adolescent Girls' Anaemia Control Programme: A decade of programming experience to break the inter‐generational cycle of malnutrition in India. Public Health Nutrition, 16, 1667–1676. 23343620 10.1017/S1368980012005587 PMC10271399

[mcn13391-bib-0003] Ahankari, A. S. , Myles, P. R. , Fogarty, A. W. , Dixit, J. V. , & Tata, L. J. (2017). Prevalence of iron‐deficiency anaemia and risk factors in 1010 adolescent girls from rural Maharashtra, India: A cross‐sectional survey. Public Health, 142, 159–166. 27592006 10.1016/j.puhe.2016.07.010

[mcn13391-bib-0004] Ahmed, F. , Khan, M. R. , Islam, M. , Kabir, I. , & Fuchs, G. J. (2000). Anaemia and iron deficiency among adolescent schoolgirls in peri‐urban Bangladesh. European Journal of Clinical Nutrition, 54, 678–83. 11002378 10.1038/sj.ejcn.1601073

[mcn13391-bib-0005] Allison, P. D. (2003). Missing data techniques for structural equation modeling. Journal of Abnormal Psychology, 112, 545–557. 14674868 10.1037/0021-843X.112.4.545

[mcn13391-bib-0006] Arsenault, J. E. , Mora‐Plazas, M. , Forero, Y. , Lopez‐Arana, S. , Baylin, A. , & Villamor, E. (2009). Hemoglobin concentration is inversely associated with erythrocyte folate concentrations in Colombian school‐age children, especially among children with low vitamin B12 status. European Journal of Clinical Nutrition, 63, 842–849. 18957973 10.1038/ejcn.2008.50

[mcn13391-bib-0007] Atasoy, H. I. , & Bugdayci, G. (2018). Zinc deficiency and its predictive capacity for anemia: Unique model in school children. Pediatrics International: Official Journal of the Japan Pediatric Society, 60, 703–709. 29804328 10.1111/ped.13603

[mcn13391-bib-0008] Azzopardi, P. S. , Hearps, S. J. C. , Francis, K. L. , Kennedy, E. C. , Mokdad, A. H. , Kassebaum, N. J. , Lim, S. , Irvine, C. M. S. , Vos, T. , Brown, A. D. , Dogra, S. , Kinner, S. A. , Kaoma, N. S. , Naguib, M. , Reavley, N. J. , Requejo, J. , Santelli, J. S. , Sawyer, S. M. , Skirbekk, V. , … Patton, G. C. (2019). Articles progress in adolescent health and wellbeing: Tracking 12 headline indicators for 195 countries and territories, 1990–2016. The Lancet, 393, 1101–1118. 10.1016/S0140-6736(18)32427-9 PMC6429986 30876706

[mcn13391-bib-0009] Balarajan, Y. , Ramakrishnan, U. , Ozaltin, E. , Shankar, A. H. , & Subramanian, S. V. (2011). Anaemia in low‐income and middle‐income countries. The Lancet, 378, 2123–2135. 10.1016/S0140-6736(10)62304-5 21813172

[mcn13391-bib-0010] Balarajan, Y. S. , Fawzi, W. W. , & Subramanian, S. V. (2013). Changing patterns of social inequalities in anaemia among women in India: Cross‐sectional study using nationally representative data. BMJ Open, 3, e002233. 10.1136/bmjopen-2012-002233 PMC3612779 23516270

[mcn13391-bib-0011] Benoist, D. (2008). Conclusions of a WHO technical consultation on folate and vitamin B 12 deficiencies. Food and Nutrition Bulletin, 29(suppl), 238–244. 10.1177/15648265080292S129 18709899

[mcn13391-bib-0012] Bharati, P. , Shome, S. , Chakrabarty, S. , Bharati, S. , & Pal, M. (2009). Burden of anemia and its socioeconomic determinants among adolescent girls in India. Food and Nutrition Bulletin, 30, 217–226. 19927601 10.1177/156482650903000302

[mcn13391-bib-0013] Bhukhanvala, D. S. , Sorathiya, S. M. , Shah, A. P. , Patel, A. G. , & Gupte, S. C. (2012). Prevalence and hematological profile of β‐thalassemia and sickle cell anemia in four communities of Surat city. Indian Journal of Human Genetics, 18, 167–171. 23162290 10.4103/0971-6866.100752 PMC3491288

[mcn13391-bib-0014] Black, R. E. , Victora, C. G. , Walker, S. P. , Bhutta, Z. A. , Christian, P. , de Onis, M. , Ezzati, M. , Grantham‐McGregor, S. , Katz, J. , Martorell, R. , Uauy, R. , & Maternal and Child Nutrition Study Group . (2013). Maternal and child undernutrition and overweight in low‐income and middle‐income countries. The Lancet, 382, 427–451. 10.1016/S0140-6736(13)60937-X 23746772

[mcn13391-bib-0015] Boghani, S. , Mei, Z. , Perry, G. S. , Brittenham, G. M. , & Cogswell, M. E. (2017). Accuracy of capillary hemoglobin measurements for the detection of anemia among U.S. low‐income toddlerstoddlers and pregnant womenpregnant women. Nutrients, 9, 253. 10.3390/nu9030253 PMC5372916 28282926

[mcn13391-bib-0016] Caicedo, O. , Villamor, E. , Forero, Y. , Ziade, J. , Pérez, P. , Quiñones, F. , Arévalo‐Herrera, M. , & Herrera, S. (2010). Relation between vitamin B12 and folate status, and hemoglobin concentration and parasitemia during acute malaria infections in Colombia. Acta Tropica, 114, 17–21. 19931503 10.1016/j.actatropica.2009.11.005 PMC2860300

[mcn13391-bib-0017] Chakrabarti, S. , George, N. , Majumder, M. , Raykar, N. , & Scott, S. (2018). Identifying sociodemographic, programmatic and dietary drivers of anaemia reduction in pregnant Indian women over 10 years. Public Health Nutrition, 21, 2424–2433. 29642966 10.1017/S1368980018000903 PMC10284710

[mcn13391-bib-0018] Chaparro, C. M. , & Suchdev, P. S. (2019). Anemia epidemiology, pathophysiology, and etiology in low‐ and middle‐income countries. Annals of the New York Academy of Sciences, 1450, 15–31. 31008520 10.1111/nyas.14092 PMC6697587

[mcn13391-bib-0019] da Cunha, M. S. B. , Siqueira, E. M. A. , Trindade, L. S. , & Arruda, S. F. (2014). Vitamin A deficiency modulates iron metabolism via ineffective erythropoiesis. The Journal of Nutritional Biochemistry, 25, 1035–1044. 24998947 10.1016/j.jnutbio.2014.05.005

[mcn13391-bib-0020] Darroch, J. E. , Woog, V. , & Bankole, A. (2016). Adding it up: Costs and benefits of meeting the contraceptive needs of adolescents. Guttmacher Institute.

[mcn13391-bib-0021] Department of Food and Public Distribution . (2020). Fortification of rice and its distribution under Public Distribution System: Operational guidelines.

[mcn13391-bib-0022] Engle‐Stone, R. , Aaron, G. J. , Huang, J. , Wirth, J. P. , Namaste, S. M. , Williams, A. M. , et al. (2017). Predictors of anemia in preschool children: Biomarkers Reflecting Inflammation and Nutritional Determinants of Anemia (BRINDA) project. The American Journal of Clinical Nutrition, 106, 402S–415S. 28615260 10.3945/ajcn.116.142323 PMC5490650

[mcn13391-bib-0023] Figueiredo, A. C. M. G. , Gomes‐Filho, I. S. , Silva, R. B. , Pereira, P. P. S. , Mata, F. A. F. D. , Lyrio, A. O. , Souza, E. S. , Cruz, S. S. , & Pereira, M. G. (2018). Maternal anemia and low birth weight: A systematic review and meta‐analysis. Nutrients, 10(5):601. 10.3390/nu10050601 PMC5986481 29757207

[mcn13391-bib-0024] Ford, N. D. , Bichha, R. P. , Parajuli, K. R. , Paudyal, N. , Joshi, N. , Whitehead, R. D., Jr. , Chitekwe, S. , Mei, Z. , Flores‐Ayala, R. , Adhikari, D. P. , Rijal, S. , & Jefferds, M. E. (2020a). Factors associated with anaemia among adolescent boys and girls 10–19 years old in Nepal. Maternal & Child Nutrition, 18(suppl 1):e13013. 32338438 10.1111/mcn.13013 PMC8770652

[mcn13391-bib-0025] Ford, N. D. , Bichha, R. P. , Parajuli, K. R. , Paudyal, N. , Joshi, N. , Whitehead, Jr., R. D. , Chitekwe, S. , Mei, Z. , Flores‐Ayala, R. , Adhikari, D. P. , Rijal, S. , & Jefferds, M. E. (2020b). Age, ethnicity, glucose‐6‐phosphate dehydrogenase deficiency, micronutrient powder intake, and biomarkers of micronutrient status, infection, and inflammation are associated with anemia among children 6–59 months in Nepal. The Journal of Nutrition, 150, 929–937. 31883009 10.1093/jn/nxz307 PMC7350881

[mcn13391-bib-0026] GBD 2017 Disease and Injury Incidence and Prevalence Collaborators . (2018). Global, regional, and national incidence, prevalence, and years lived with disability for 354 diseases and injuries for 195 countries and territories, 1990–2017: A systematic analysis for the Global Burden of Disease Study 2017. Lancet, 392, 1789–1858. 30496104 10.1016/S0140-6736(18)32279-7 PMC6227754

[mcn13391-bib-0027] George, J. , Yiannakis, M. , Main, B. , Devenish, R. , Anderson, C. , An, U. S. , Williams, S. M. , & Gibson, R. S. (2012). Genetic hemoglobin disorders, infection, and deficiencies of iron and vitamin A determine anemia in young Cambodian children. The Journal of Nutrition, 142, 781–787. 22378325 10.3945/jn.111.148189 PMC3301994

[mcn13391-bib-0028] Gwetu, T. P. , Chhagan, M. , Craib, M. , & Kauchali, S. (2013). Hemocue validation for the diagnosis of anaemia in children: A semi systematic review. Pediatric Therapeutics, 4, 1.

[mcn13391-bib-0029] Haas, J. D. , & Brownlie, T. (2001). Iron deficiency and reduced work capacity: A critical review of the research to determine a causal relationship. The Journal of Nutrition, 131, 676S–688S; discussion 688S‐690S. 11160598 10.1093/jn/131.2.676S

[mcn13391-bib-0030] Haider, B. A. , Olofin, I. , Wang, M. , Spiegelman, D. , Ezzati, M. , & Fawzi, W. W. (2013). Anaemia, prenatal iron use, and risk of adverse pregnancy outcomes: Systematic review and meta‐analysis. BMJ (Clinical research ed.), 346, f3443. 10.1136/bmj.f3443 PMC3689887 23794316

[mcn13391-bib-0031] Hargreaves, D. , Mates, E. , Menon, P. , Alderman, H. , Devakumar, D. , Fawzi, W. , Greenfield, G. , Hammoudeh, W. , He, S. , Lahiri, A. , Liu, Z. , Nguyen, P. H. , Sethi, V. , Wang, H. , Neufeld, L. M. , & Patton, G. C. (2021). Strategies and interventions for healthy adolescent growth, nutrition, and development. *The Lancet*, 399(10320), 198–210. 10.1016/S0140-6736(21)01593-2 34856192

[mcn13391-bib-0032] Houghton, L. A. , Parnell, W. R. , Thomson, C. D. , Green, T. J. , & Gibson, R. S. (2016). Serum zinc is a major predictor of anemia and mediates the effect of selenium on hemoglobin in school‐aged children in a nationally representative survey in New Zealand. The Journal of Nutrition, 146, 1670–1676. 27466609 10.3945/jn.116.235127

[mcn13391-bib-0033] International Institute for Population Sciences . (2022). India Fact Sheet. *National Family Health Survey‐5 2019‐21*. Ministry of Health and Family Welfare, Government of India.

[mcn13391-bib-0034] Karakochuk, C. D. , Whitfield, K. C. , Barr, S. I. , Lamers, Y. , Devlin, A. M. , Vercauteren, S. M. , Kroeun, H. , Talukder, A. , McLean, J. , & Green, T. J. (2015). Genetic hemoglobin disorders rather than iron deficiency are a major predictor of hemoglobin concentration in women of reproductive age in rural prey Veng, Cambodia. The Journal of Nutrition, 145, 134–142. 25527668 10.3945/jn.114.198945

[mcn13391-bib-0035] Madan, N. , Sharma, S. , Sood, S. K. , Colah, R. , & Bhatia, L. H. M. (2010). Frequency of β‐thalassemia trait and other hemoglobinopathies in Northern and Western India. Indian Journal of Human Genetics, 16, 16–25. 20838487 10.4103/0971-6866.64941 PMC2927789

[mcn13391-bib-0036] McGann, P. T. , Williams, A. M. , Ellis, G. , McElhinney, K. E. , Romano, L. , Woodall, J. , Howard, T. A. , Tegha, G. , Krysiak, R. , Lark, R. M. , Ander, E. L. , Mapango, C. , Ataga, K. I. , Gopal, S. , Key, N. S. , Ware, R. E. , & Suchdev, P. S. (2018). Prevalence of inherited blood disorders and associations with malaria and anemia in Malawian children. Blood Advances, 2, 3035–3044. 30425067 10.1182/bloodadvances.2018023069 PMC6234379

[mcn13391-bib-0037] Merrill, R. D. , Burke, R. M. , Northrop‐Clewes, C. A. , Rayco‐Solon, P. , Flores‐Ayala, R. , Namaste, S. M. , Serdula, M. K. , & Suchdev, P. S. (2017). Factors associated with inflammation in preschool children and women of reproductive age: Biomarkers Reflecting Inflammation and Nutritional Determinants of Anemia (BRINDA) project. The American Journal of Clinical Nutrition, 106, 348S–358S. 28615263 10.3945/ajcn.116.142315 PMC5490646

[mcn13391-bib-0038] Metz, J. (2008). A high prevalence of biochemical evidence of vitamin B12 or folate deficiency does not translate into a comparable prevalence of anemia. Food and Nutrition Bulletin, 29(2, suppl), 74–85. 10.1177/15648265080292S111 18709883

[mcn13391-bib-0039] Ministry of Health and Family Welfare . (2016). National Health Mission—Guidelines on hemoglobinopathies in India: Prevention and control of hemoglobinopathies in India.

[mcn13391-bib-0040] Ministry of Health and Family Welfare, Government of India . (2012). Operational Framework. Weekly iron and folic acid supplementation programme for adolescents.

[mcn13391-bib-0041] Ministry of Health and Family Welfare, Government of India . (2018). Intensified National Iron Plus Initiative (I‐NIPI). Operational Guidelines for Programme Managers.

[mcn13391-bib-0042] Ministry of Health and Family Welfare, Government of India . (2019). National Family Health Survey (NFHS‐5), Key Indicators.

[mcn13391-bib-0043] Ministry of Health and Family Welfare (MoHFW), Government of India, UNICEF, & Population Council . (2019). *Comprehensive National Nutrition Survey (CNNS)* (National Report).

[mcn13391-bib-0044] Mohanty, D. , Gorakshakar, A. C. , Colah, R. B. , Patel, R. Z. , Master, D. C. , Mahanta, J. , Sharma, S. K. , Chaudhari, U. , Ghosh, M. , Das, S. , Britt, R. P. , Singh, S. , Ross, C. , Jagannathan, L. , Kaul, R. , Shukla, D. K. , & Muthuswamy, V. (2014). Interaction of iron deficiency anemia and hemoglobinopathies among college students and pregnant women: A multi‐center evaluation in India. Hemoglobin, 38, 252–257. 25023086 10.3109/03630269.2014.913517

[mcn13391-bib-0045] Mohanty, D. , Mukherjee, M. B. , Colah, R. B. , Wadia, M. , Ghosh, K. , Chottray, G. P. , Jain, D. , Italia, Y. , Ashokan, K. , Kaul, R. , Shukla, D. K. , & Muthuswamy, V. (2008). Iron deficiency anaemia in sickle cell disorders in India. The Indian Journal of Medical Research, 127, 366–369. 18577791

[mcn13391-bib-0046] Morris, M. S. , Jacques, P. F. , Rosenberg, I. H. , & Selhub, J. (2007). Folate and vitamin B‐12 status in relation to anemia, macrocytosis, and cognitive impairment in older Americans in the age of folic acid fortification. American Journal of Clinical Nutrition, 85, 193–200. 17209196 10.1093/ajcn/85.1.193 PMC1828842

[mcn13391-bib-0047] Mukherjee, S. B. (2016). Growth, nutritional status and anemia in Indian adolescents. Indian Pediatrics, 53, 905–906. 27771671 10.1007/s13312-016-0956-3

[mcn13391-bib-0048] Namaste, S. M. , Aaron, G. J. , Varadhan, R. , Peerson, J. M. , & Suchdev, P. S. (2017). Methodologic approach for the Biomarkers Reflecting Inflammation and Nutritional Determinants of Anemia (BRINDA) project. The American Journal of Clinical Nutrition, 106(suppl 1), 333S–347S. 28615254 10.3945/ajcn.116.142273 PMC5490643

[mcn13391-bib-0049] National Commission on Population, Ministry of Health and Family Welfare . (2020). *Population projections for India and States 2011–2036*.

[mcn13391-bib-0050] Nguyen, P. H. , Gonzalez‐Casanova, I. , Nguyen, H. , Pham, H. , Truong, T. V. , Nguyen, S. , Martorell, R. , & Ramakrishnan, U. (2015). Multicausal etiology of anemia among women of reproductive age in Vietnam. European Journal of Clinical Nutrition, 69, 107–13. 25205323 10.1038/ejcn.2014.181

[mcn13391-bib-0051] Nguyen, P. H. , Scott, S. , Avula, R. , Tran, L. M. , & Menon, P. (2018). Trends and drivers of change in the prevalence of anaemia among 1 million women and children in India, 2006 to 2016. BMJ Global Health, 3, e001010. 10.1136/bmjgh-2018-001010 PMC6202996 30397516

[mcn13391-bib-0052] Pasricha, S.‐R. , Black, J. , Muthayya, S. , Shet, A. , Bhat, V. , Nagaraj, S. , Prashanth, N. S. , & Sudarshan, H. (2010). Determinants of anemia among young children in rural India. Pediatrics, 126(1), e140–e149. 10.1542/peds.2009-3108 20547647

[mcn13391-bib-0053] Patel, S. , Dhuppar, P. , & Bhattar, A. (2017). Nutritional anemia status in adolescent girls in rural schools of Raipur, India. Medicinal Chemistry, 7, 4.

[mcn13391-bib-0054] Patra, P. K. , Khodiar, P. K. , Hambleton, I. R. , & Serjeant, G. R. (2015). The Chhattisgarh state screening programme for the sickle cell gene: A cost‐effective approach to a public health problem. Journal of Community Genetics, 6, 361–368. 25822801 10.1007/s12687-015-0222-8 PMC4567986

[mcn13391-bib-0055] Petry, N. , Jallow, B. , Sawo, Y. , Darboe, M. K. , Barrow, S. , Sarr, A. , Ceesay, P. O. , Fofana, M. N. , Prentice, A. M. , Wegmüller, R. , Rohner, F. , Phall, M. C. , & Wirth, J. P. (2019). Micronutrient deficiencies, nutritional status and the determinants of anemia in children 0–59 months of age and non‐pregnant women of reproductive age in The Gambia. Nutrients, 11, 2275. 10.3390/nu11102275 PMC6835426 31547543

[mcn13391-bib-0056] Prieto‐Patron, A. , Van der Horst, K. , Hutton, Z. V. , & Detzel, P. (2018). Association between anaemia in children 6 to 23 months old and child, mother, household and feeding indicators. Nutrients, 10, 1269. 10.3390/nu10091269 PMC6163758 30205553

[mcn13391-bib-0057] Qiu, A. , Jansen, M. , Sakaris, A. , Min, S. H. , Chattopadhyay, S. , Tsai, E. , Sandoval, C. , Zhao, R. , Akabas, M. H. , & Goldman, I. D. (2006). Identification of an intestinal folate transporter and the molecular basis for hereditary folate malabsorption. Cell, 127, 917–928. 17129779 10.1016/j.cell.2006.09.041

[mcn13391-bib-0058] Ramakrishnan, U. , Lowe, A. , Vir, S. , Kumar, S. , Mohanraj, R. , Chaturvedi, A. , Noznesky, E. A. , Martorell, R. , & Mason, J. B. (2012). Public health interventions, barriers, and opportunities for improving maternal nutrition in India. Food and Nutrition Bulletin, 33(2, suppl), S71–S92. 22913108 10.1177/15648265120332S105

[mcn13391-bib-0059] Rogers, L. M. , Boy, E. , Miller, J. W. , Green, R. , Sabel, J. C. , & Allen, L. H. (2003). High prevalence of cobalamin deficiency in Guatemalan schoolchildren: Associations with low plasma holotranscobalamin II and elevated serum methylmalonic acid and plasma homocysteine concentrations. American Journal of Clinical Nutrition, 77, 433–440. 12540405 10.1093/ajcn/77.2.433

[mcn13391-bib-0060] Rustein, S. O. , & Johnson, K. (2004). The DHS Wealth Index. DHS Comparative Reports No. 6. ORC Macro.

[mcn13391-bib-0061] Sachdev, H. S. , Porwal, A. , Acharya, R. , Ashraf, S. , Ramesh, S. , Khan, N. , Kapil, U. , Kurpad, A. V. , & Sarna, A. (2021). Haemoglobin thresholds to define anaemia in a national sample of healthy children and adolescents aged 1–19 years in India: A population‐based study. The Lancet Global Health, 9, e822–e831. 33872581 10.1016/S2214-109X(21)00077-2 PMC7612991

[mcn13391-bib-0062] Saraya, A. K. , Choudhry, V. P. , & Ghai, O. P. (1973). Interrelationships of vitamin B 12, folic acid, and iron in anemia of infancy and childhood: Effect of vitamin B 12 and iron therapy on folate metabolism. American Journal of Clinical Nutrition, 26, 640–646. 4707970 10.1093/ajcn/26.6.640

[mcn13391-bib-0063] Sarna, A. , Porwal, A. , Ramesh, S. , Agrawal, P. , Acharya, R. , Johnston, R. , Khan, N. , Sachdev, H. P. S. , Nair, K. M. , Ramakrishnan, L. , Abraham, R. , Deb, S. , Khera, A. , & Saxena, R. (2020). Characterisation of the types of anaemia prevalent among children and adolescents aged 1–19 years in India: A population‐based study. The Lancet Child & Adolescent Health, 4, 515–525. 32562633 10.1016/S2352-4642(20)30094-8

[mcn13391-bib-0064] Scott, S. P. , Murray‐Kolb, L. E. , Wenger, M. J. , Udipi, S. A. , Ghugre, P. S. , Boy, E. , & Haas, J. D. (2018). Cognitive performance in Indian school‐going adolescents is positively affected by consumption of iron‐biofortified pearl millet: A 6‐month Randomized Controlled Efficacy Trial. The Journal of Nutrition, 148, 1462–1471. 30016516 10.1093/jn/nxy113

[mcn13391-bib-0065] Semba, R. D. , & Bloem, M. W. (2002). The anemia of vitamin A deficiency: Epidemiology and pathogenesis. European Journal of Clinical Nutrition, 56, 271–281. 11965502 10.1038/sj.ejcn.1601320

[mcn13391-bib-0066] Sethi, V. , Sternin, M. , Sharma, D. , Bhanot, A. , & Mebrahtu, S. (2017). Applying positive deviance for improving compliance to Adolescent Anemia Control Program in tribal communities of India. Food and Nutrition Bulletin, 38, 447–452. 28748723 10.1177/0379572117712791

[mcn13391-bib-0067] Shaban, L. , Al‐taiar, A. , Rahman, A. , & Al‐sabah, R. (2020). Anemia and its associated factors among adolescents in Kuwait. Scientific Reports, 10, 1–9. 32246050 10.1038/s41598-020-60816-7 PMC7125127

[mcn13391-bib-0068] Shayeghi, M. , Latunde‐Dada, G. O. , Oakhill, J. S. , Laftah, A. H. , Takeuchi, K. , Halliday, N. , Khan, Y. , Warley, A. , McCann, F. E. , Hider, R. C. , Frazer, D. M. , Anderson, G. J. , Vulpe, C. D. , Simpson, R. J. , & McKie, A. T. (2005). Identification of an intestinal heme transporter. Cell, 122, 789–801. 16143108 10.1016/j.cell.2005.06.025

[mcn13391-bib-0069] Srivastava, A. , Kumar, R. , & Sharma, M. (2016). Nutritional anaemia in adolescent girls: An epidemiological study. International Journal of Community Medicine and Public Health, 3, 808–812.

[mcn13391-bib-0070] Thomas, D. , Chandra, J. , Sharma, S. , Jain, A. , & Pemde, H. K. (2015). Determinants of nutritional anemia in adolescents. Indian Pediatrics, 52, 867–869. 26499011 10.1007/s13312-015-0734-7

[mcn13391-bib-0071] Toteja, G. S. , Singh, P. , Dhillon, B. S. , Saxena, B. N. , Ahmed, F. U. , Singh, R. P. , Prakash, B. , Vijayaraghavan, K. , Singh, Y. , Rauf, A. , Sarma, U. C. , Gandhi, S. , Behl, L. , Mukherjee, K. , Swami, S. S. , Meru, V. , Chandra, P. , Chandrawati, Chandrawathi. , & Mohan, U. (2006). Prevalence of anemia among pregnant women and adolescent girls in 16 districts of India. Food and Nutrition Bulletin, 27, 311–315. 17209473 10.1177/156482650602700405

[mcn13391-bib-0072] UNICEF . (2018). Forging an anemia‐free future: The path to India's nationwide adolescent anaemia control programme.

[mcn13391-bib-0073] Varghese, J. S. , & Stein, A. D. (2019). Malnutrition among women and children in India: Limited evidence of clustering of underweight, anemia, overweight, and stunting within individuals and households at both state and district levels. The American Journal of Clinical Nutrition, 109, 1207–15. 30882139 10.1093/ajcn/nqy374

[mcn13391-bib-0074] Vrieze, S. I. (2012). Model selection and psychological theory: A discussion of the differences between the Akaike information criterion (AIC) and the Bayesian information criterion (BIC). Psychological Methods, 17, 228–243. 22309957 10.1037/a0027127 PMC3366160

[mcn13391-bib-0075] Whitehead, R. D., Jr. , Mei, Z. , Mapango, C. , & Jefferds, M. E. D. (2019). Methods and analyzers for hemoglobin measurement in clinical laboratories and field settings. Annals of the New York Academy of Sciences, 1450, 147–171. 31162693 10.1111/nyas.14124 PMC6709845

[mcn13391-bib-0076] WHO . (2011a). Haemoglobin concentrations for the diagnosis of anaemia and assessment of severity.

[mcn13391-bib-0077] WHO. (2011b). Serum ferritin concentrations for the assessment of iron status and iron deficiency in populations.

[mcn13391-bib-0078] WHO . (2017). Nutritional anaemias: Tools for effective prevention.

[mcn13391-bib-0079] Wirth, J. P. , Woodruff, B. A. , Engle‐Stone, R. , Namaste, S. M. , Temple, V. J. , Petry, N. , Macdonald, B. , Suchdev, P. S. , Rohner, F. , & Aaron, G. J. (2017). Predictors of anemia in women of reproductive age: Biomarkers Reflecting Inflammation and Nutritional Determinants of Anemia (BRINDA) project. The American Journal of Clinical Nutrition, 106(suppl 1), 416S–427S. 28615262 10.3945/ajcn.116.143073 PMC5490645

