# Supplementary material for: Anaemia in Indians aged 10–19 years: Prevalence, burden and associated factors at national and regional levels
Source: Matern Child Nutr. 2022 Jun 20;18(4):e13391. doi: 10.1111/mcn.13391 (PMC9480897; doi:10.1111/mcn.13391)
Supplement: Supplementary file 1 — Supporting information. [file MCN-18-e13391-s001.docx]

**Supplementary Tables and Figures**

| **Table S1. Odds of anemia by proximate and distal factors in Indian adolescents aged 10-19 years, results from national-level multivariable logistic regression models** | | | | | | | | | |  |  |  |  |
| --- | --- | --- | --- | --- | --- | --- | --- | --- | --- | --- | --- | --- | --- |
|  | | **Bivariate** | | **Multivariate** | | **Multivariate** | | **Multivariate** | | **Multivariate** | | | |
|  | |  | | **Proximate factors** | | **Distal factors** | | **Overall-**  **exclude high CRP** | | **Overall-**  **include high CRP** | | | |
|  | |  |  |  | |  | | **n= 6156** | | **n= 6781** | | | |
| **Proximate factors** | | *OR [95% CI]* | | *OR [95% CI]* | | *OR [95% CI]* | | *OR [95% CI]* | | *OR [95% CI]* | | | |
| Dietary factors | |  |  |  |  |  |  |  |  |  | | |  |
| Consumed ASF (weekly) | | 0.85 | [0.72,1.01] | 1.01 | [0.78,1.31] |  |  | 1.16 | [0.87,1.56] | 1.17 | | | [0.89,1.53] |
| Consumed deworming tab (last 6m) | | 0.86 | [0.72,1.02] | 0.84 | [0.65,1.09] |  |  | 1.11 | [0.84,1.46] | 1.07 | | | [0.83,1.39] |
| Consumed IFA supplements (last 1wk) | | 1.2 | [0.94,1.52] | 1.3 | [0.90,1.87] |  |  | 1.18 | [0.80,1.72] | 1.22 | | | [0.85,1.74] |
| Micronutrient deficiencies | |  |  |  |  |  |  |  |  |  | | |  |
| Iron deficiency | | 4.59*** | [3.72,5.67] | 4.96*** | [3.58,6.87] |  |  | 4.68*** | [3.21,6.83] | 4.36*** | | | [3.02,6.31] |
| Vitamin B12 deficiency | | 0.81 | [0.66,1.00] | 0.8 | [0.57,1.13] |  |  | 0.99 | [0.68,1.43] | 0.98 | | | [0.69,1.40] |
| Folate deficiency | | 0.67*** | [0.57,0.80] | 0.49*** | [0.38,0.64] |  |  | 0.60*** | [0.46,0.80] | 0.59*** | | | [0.46,0.76] |
| Vitamin A deficiency | | 1.25 | [0.92,1.69] | 1.74** | [1.19,2.55] |  |  | 1.86** | [1.23,2.80] | 1.70** | | | [1.15,2.51] |
| Vitamin D deficiency | | 1.47*** | [1.22,1.77] | 1.23 | [0.94,1.62] |  |  | 0.95 | [0.70,1.28] | 0.96 | | | [0.72,1.27] |
| Zinc Deficiency | | 1.22* | [1.01,1.47] | 1.18 | [0.91,1.53] |  |  | 1.32* | [1.02,1.72] | 1.31* | | | [1.02,1.69] |
| Hemoglobinopathies | | 2.93*** | [2.14,4.03] | 3.06*** | [1.88,4.98] |  |  | 2.81*** | [1.66,4.74] | 2.77*** | | | [1.70,4.50] |
| **Distal factors** | |  |  |  |  |  |  |  |  |  | | |  |
| Sociodemographic factors | |  |  |  |  |  |  |  |  |  | | |  |
| Aged 15-19 years (ref 10-14 years) | | 1.55*** | [1.30,1.84] |  |  | 1.38** | [1.12,1.71] | 1.57* | [1.10,2.25] | 1.54* | | | [1.10,2.16] |
| Female | | 3.12*** | [2.62,3.72] |  |  | 3.13*** | [2.62,3.73] | 2.56*** | [1.94,3.39] | 2.62*** | | | [2.01,3.41] |
| Currently in school | | 0.66*** | [0.54,0.81] |  |  | 0.88 | [0.69,1.12] | 0.87 | [0.58,1.30] | 0.93 | | | [0.63,1.36] |
| Wealth Index (ref: richest) | |  |  |  |  |  |  |  |  |  | | |  |
| Poorest | | 1.59*** | [1.21,2.09] |  |  | 0.98 | [0.63,1.51] | 0.91 | [0.43,1.91] | 1.19 | | | [0.59,2.42] |
| Poor | | 1.48** | [1.14,1.91] |  |  | 1.04 | [0.72,1.51] | 1.18 | [0.66,2.11] | 1.42 | | | [0.81,2.49] |
| Middle | | 1.36* | [1.07,1.74] |  |  | 1.08 | [0.81,1.46] | 1.11 | [0.71,1.73] | 1.21 | | | [0.79,1.85] |
| Rich | | 1.46** | [1.13,1.89] |  |  | 1.22 | [0.93,1.60] | 1.22 | [0.76,1.95] | 1.26 | | | [0.82,1.94] |
| Either parent illiterate (ref: both literate) | | 1.23* | [1.04,1.46] |  |  | 1.09 | [0.90,1.33] | 1.13 | [0.82,1.54] | 1.11 | | | [0.83,1.49] |
| Environmental factors | |  |  |  |  |  |  |  |  |  | | |  |
| Access to improved sanitation | | 0.87 | [0.73,1.03] |  |  | 0.94 | [0.75,1.18] | 0.95 | [0.69,1.32] | 1.02 | | | [0.74,1.40] |
| Access to soap and water for handwash | | 0.83* | [0.70,0.99] |  |  | 0.94 | [0.77,1.15] | 0.92 | [0.65,1.28] | 0.99 | | | [0.72,1.37] |
| Mass Media exposure level (ref: high) | |  |  |  |  |  |  |  |  |  | | |  |
| Low | | 1.61** | [1.17,2.22] |  |  | 1.32 | [0.96,1.82] | 1.04 | [0.67,1.61] | 1.08 | | | [0.72,1.63] |
| Medium | | 1.44* | [1.03,2.02] |  |  | 1.3 | [0.93,1.82] | 1.02 | [0.63,1.66] | 1.06 | | | [0.68,1.65] |
| Received mid-day meal in school | | 0.75** | [0.62,0.90] |  |  | 0.8 | [0.62,1.03] | 0.88 | [0.60,1.29] | 0.78 | | | [0.54,1.13] |
| * p<0.05, ** p<0.01, *** p<0.001 from logistic multivariable regression models. Separate models were run for boys, girls and overall (sexes combined). All models controlled for residence (rural/urban), religion, caste and region. ref = reference category. ASF, animal sourced foods; IFA, iron folic acid | | | | | | | | | |  | | | |

| **Table** **S2. Prevalence of anemia in adolescents by state, CNNS 2016-18** | | | | | | | | | | |  | |
| --- | --- | --- | --- | --- | --- | --- | --- | --- | --- | --- | --- | --- |
|  | **Boys** | | | **Girls** | | | **Overall** | | | |  | |
|  | *10-14y* | *15-19y* | *10-19y* | *10-14y* | *15-19y* | *10-19y* | *10-14y* | *15-19y* | *10-19y* |  | |  |
| N | 4081 | 3508 | 7589 | 3727 | 3353 | 7080 | 6861 | 7808 | 14669 | Overall N (10-19y) | |  |
| **India** | **17.1** | **18.3** | **17.6** | **32.3** | **47.5** | **39.6** | **24.5** | **33** | **28.5** | **14669** | |  |
| **North** | | | | | | | | | | |  | |
| Delhi | 12.5 | 18.8 | 15.6 | 33.4 | 56.5 | 45.8 | 21.6 | 37.1 | 29.5 | 640 | |  |
| Haryana | 23.2 | 20.5 | 21.7 | 29.5 | 54.2 | 40.7 | 26.2 | 33.4 | 29.9 | 489 | |  |
| Himachal Pradesh | 4.4 | 20.9 | 12.8 | 12.1 | 28.2 | 19.1 | 8.8 | 24.6 | 16.2 | 430 | |  |
| Jammu and Kashmir | 6.7 | 5.6 | 6.3 | 15.6 | 31.9 | 23.8 | 11.1 | 21.5 | 15.8 | 336 | |  |
| Punjab | 16.6 | 13.7 | 15.1 | 24.1 | 46.9 | 36.9 | 20.2 | 30.8 | 25.9 | 496 | |  |
| Rajasthan | 6 | 17.1 | 11.2 | 31.1 | 50.4 | 40.1 | 18.9 | 34.2 | 26 | 528 | |  |
| Uttarakhand | 6.8 | 15.7 | 11.7 | 17 | 23.7 | 20.2 | 12 | 19.1 | 15.7 | 467 | |  |
| **Central** | | | | | | | | | | |  | |
| Chhattisgarh | 20.4 | 21.2 | 20.7 | 41.7 | 41.5 | 41.6 | 30.9 | 31.7 | 31.3 | 480 | |  |
| Madhya Pradesh | 12.1 | 19.7 | 15.4 | 23.3 | 34.1 | 28.7 | 16.7 | 26.5 | 21.2 | 501 | |  |
| Uttar Pradesh | 17.9 | 16.8 | 17.4 | 40.8 | 49.4 | 45.1 | 29.3 | 34.7 | 31.9 | 472 | |  |
| **East** | | | | | | | | | | |  | |
| Bihar | 20.5 | 16.4 | 18.7 | 23 | 46 | 35.6 | 21.7 | 34.5 | 28 | 654 | |  |
| Jharkhand | 17.5 | 14.8 | 16.4 | 50.5 | 53.6 | 51.8 | 33.9 | 34.3 | 34 | 508 | |  |
| Odisha | 18.4 | 18.3 | 18.3 | 31 | 50.8 | 40.8 | 24.8 | 34.7 | 29.6 | 696 | |  |
| West Bengal | 31.4 | 28 | 29.8 | 56.1 | 67.6 | 62 | 43.1 | 48.3 | 45.6 | 700 | |  |
| **Northeast** | | | | | | | | | | |  | |
| Arunachal Pradesh | 20.5 | 17.8 | 19.2 | 22 | 48.1 | 34.6 | 21.3 | 31.8 | 26.4 | 520 | |  |
| Assam | 24.8 | 39 | 32 | 35 | 53.4 | 42.5 | 30.1 | 45.1 | 37 | 474 | |  |
| Manipur | 7.3 | 12.6 | 9.7 | 8.1 | 14.8 | 11.3 | 7.7 | 13.7 | 10.6 | 653 | |  |
| Meghalaya | 23.9 | 20.6 | 22.4 | 32.3 | 59.9 | 43.4 | 27.9 | 36.8 | 31.8 | 304 | |  |
| Mizoram | 14.1 | 9 | 11.5 | 16 | 35.7 | 24.9 | 15.1 | 21.4 | 18.1 | 368 | |  |
| Nagaland | 15.3 | 0 | 9.6 | 9.3 | 5.8 | 7.3 | 13 | 3.6 | 8.4 | 196 | |  |
| Sikkim | 17.7 | 10 | 13.7 | 30.7 | 44.7 | 37.5 | 24.4 | 27.1 | 25.8 | 580 | |  |
| Tripura | 25.7 | 33 | 29.3 | 41.3 | 67.1 | 54.5 | 33.1 | 49.6 | 41.4 | 346 | |  |
| **West** | | | | | | | | | | |  | |
| Goa | 5.8 | 6.2 | 6.1 | 14.1 | 32.1 | 22.7 | 10 | 17 | 13.6 | 342 | |  |
| Gujarat | 22.8 | 20.3 | 21.6 | 44.2 | 47.5 | 46 | 33.3 | 34.2 | 33.7 | 465 | |  |
| Maharashtra | 20.8 | 21.1 | 20.9 | 27.2 | 48.1 | 38.4 | 23.5 | 32.4 | 28.3 | 808 | |  |
| **South** | | | | | | | | | | |  | |
| Andhra Pradesh | 9.8 | 13.3 | 11.7 | 28 | 39.4 | 33.3 | 18.5 | 24 | 21.3 | 546 | |  |
| Karnataka | 4.8 | 13.5 | 9.2 | 16.6 | 35.1 | 25.6 | 10.9 | 24 | 17.4 | 348 | |  |
| Kerala | 3 | 5.2 | 4.1 | 6.5 | 21.3 | 13.7 | 4.8 | 13.6 | 9.2 | 348 | |  |
| Tamil Nadu | 7.9 | 8.1 | 8 | 14.3 | 39 | 26.4 | 11 | 22.3 | 16.7 | 546 | |  |
| Telangana | 16.7 | 20.3 | 18.5 | 37.8 | 55.1 | 46 | 27.4 | 37.1 | 32.1 | 342 | |  |
| All numbers in table are percentages; anemia prevalence is based on standard sex- and age-specific hemoglobin cutoffs recommended by the World Health Organization (see Table S1) | | | | | | | | | | |  | |
